# Dynamic interplay of immune response, metabolome, and microbiota in cows during high-grain feeding: insights from multi-omics analysis

**DOI:** 10.1128/spectrum.00944-24

**Published:** 2024-08-20

**Authors:** Ezequias Castillo-Lopez, Sara Ricci, Raul Rivera-Chacon, Arife Sener-Aydemir, Cátia Pacífico, Nicole Reisinger, Heidi E. Schwartz-Zimmermann, Franz Berthiller, Susanne Kreuzer-Redmer, Qendrim Zebeli

**Affiliations:** 1 Center for Animal Nutrition and Welfare, Clinical Department for Farm Animals and Food System Science, University of Veterinary Medicine Vienna, Vienna, Austria; 2 Christian Doppler Laboratory for Innovative Gut Health Concepts of Livestock, Vienna, Austria; 3 Biome Diagnostics GmbH, Vienna, Austria; 4 Unit of Food Hygiene and Technology, Institute of Food Safety, Food Technology and Veterinary Public Health, University of Veterinary Medicine Vienna, Vienna, Austria; 5 dsm-firmenich, Animal Nutrition and Health R&D Center, Tulln, Austria; 6 Department of Agrobiotechnology (IFA-Tulln), Institute of Bioanalytics and Agro-Metabolomics, University of Natural Resources and Life Sciences, Vienna, Austria; USDA-ARS Arkansas Children's Nutrition Center, Little Rock, Arkansas, USA

**Keywords:** immune response, gut microbiota, gene expression, metabolome, cattle

## Abstract

This study explores the dynamics of immune gene expression, ruminal metabolome, and gut microbiota in cows due to the duration of high-grain feeding, shedding light on host response and microbial dynamics in parallel. Cows consumed forage for a week, then gradually transitioned to a high-grain diet, which they consumed for 4 weeks. Immune response was evaluated in ruminal papillae by expression of genes related to the nuclear factor-kappaB (NFkB) pathway and correlated with the microbiota. Rumen metabolome was evaluated with high-performance liquid chromatography coupled with mass spectrometry and anion-exchange chromatography. Rumen and fecal microbiota were evaluated with 16S rRNA gene amplicon sequencing. In the rumen, expression of inflammation-associated genes increased with the duration on high grain, indicating activation of pro-inflammatory cascades; microbial diversity decreased with a high-grain diet but stabilized after week 3 on high grain. Changes in microbial relative abundance and metabolite enrichment were observed throughout the 4 weeks on high grain, with increments in propionogenic taxa (i.e., *Succinivibrionaceae*). Metabolite enrichment analysis showed that at the start of high-grain feeding, simple carbohydrates were enriched; then, these were substituted by their fermentation products. There were correlations between certain ruminal bacterial taxa (i.e., *Ruminococcaceae* UCG-005) and expression of genes of the NFkB pathway, suggesting the influence of these taxa on host immune response. In feces, microbial diversity and several *Ruminococcaceae* members initially declined but recovered by weeks 3 and 4. Overall, despite the stabilization of microbial diversity, changes in microbial relative abundance and proinflammatory genes were observed throughout high-grain feeding, suggesting that cows need more than 4 weeks to fully adjust once consuming a high-grain diet.

**IMPORTANCE:**

Despite the stepwise diet transition typically assumed to serve for animal adaptation, expression of signaling receptors, mediators, and downstream targets of nuclear factor-kappaB pathway were found throughout the 4 weeks on high grain, which correlated with changes in the rumen microbial profile. In addition, although microbial diversity recovered in the feces and stabilized in the rumen in week 3 on high grain, we observed changes in microbial relative abundance throughout the 4 weeks on high grain, suggesting that cows need more than 4 weeks to adjust once consuming this diet. Findings are particularly important to consider when planning experiments involving dietary changes.

## INTRODUCTION

In cattle, it has been generally perceived that during a diet transition, major changes occur with regard to microbial profiles (1), gastrointestinal fermentation (2), and expression of genes associated with nutrient utilization (3). Therefore, this timeframe has been suggested to be highly risky for cattle health (4, 5). However, the microbial- and immune-related changes due to the increased duration of high-grain feeding after the diet transition remain poorly understood, thus representing important research gaps that need to be addressed.

Evaluating the effect of increased duration of high-grain feeding on microbial and immune changes is crucial to understand the time needed for animal adjustment to a diet change. In practical terms, this knowledge may have important applications. For example, in switch-over experiments with dairy cattle, researchers have typically assumed that a timeframe of around 2 weeks is sufficient for animal adjustment after the diet change (6–9). Yet, there is no clear quantitative evidence showing that this interval is sufficient for adequate gut microbial and immune adaptation. This information is essential when switching from diets with different chemical composition.

The gastrointestinal microbiota of cattle has a vital contribution to production and health. For example, the rumen microbial community associated with the solid feed particles constitutes the majority of total bacterial biomass (10). This community plays crucial roles in key aspects such as fiber degradation and energy production (11), metabolizable protein supply (12), fatty acid biohydrogenation (13), and host immune regulation (14). Likewise, the microbiota of the hindgut influences the host immune response (15, 16) and participates in feed degradation (17). Nonetheless, both ruminal and hindgut microbiota are strongly affected by changes in dietary composition. For example, reductions in bacterial diversity have been reported in the rumen (18) and hindgut (19) due to diet changes. In addition, the change to high-grain feeding impacts the expression of genes involved in local or systemic inflammation (20, 21).

A previous study from our group, aiming to evaluate the effects of phytogenic supplementation on the rumen microbiome and epithelium during high-grain feeding, has shown that the ruminal environment could potentially adapt to a dietary change over a period of 4 weeks (22). However, the lower gut was not evaluated, leaving open the question if the adaptive process observed was limited only to the rumen. Furthermore, some of the genes involved in the nuclear factor-kappaB (NFkB) pathway, which is deemed as one of the major triggers for the development of rumenitis in association with subacute ruminal acidosis, also showed signs of adaptation to the high-grain feeding after 4 weeks (22, 23). In particular, the release of pro-inflammatory molecules triggers immune response and inflammation in relation to the levels of lipopolysaccharide (LPS) (24, 25). However, evaluating these changes due to prolonged duration of high-grain feeding is lacking in the scientific literature.

Therefore, this study aimed to evaluate the effect of duration of high-grain feeding on the dynamics of ruminal and hindgut microbiota, ruminal metabolome, and expression of inflammation-associated genes focusing on the NFkB pathway. We also aimed to evaluate correlations between the host immune response and the changes in the gut microbiota composition. We hypothesized that after the gradual diet transition, a 4-week period of high-grain feeding would be enough for cows’ gastrointestinal tract microbiome and immune response to completely adjust to the diet.

## RESULTS

### Ruminal pH variation due to the change from forage to high-grain feeding

There was a strong decline in ruminal pH due to the change from forage to high-grain feeding (Table S1 in the supplementary material). Mean ruminal pH declined by approximately 0.44 units and the time below pH 5.8 increased by 226 min/day. All data used in the present study originated from cows that remained healthy during the entire experiment and were not treated with any antibiotics. It is also worth noting that in the second experimental period, one cow experienced health complications not related to dietary management; thus, this cow was excluded from the second experimental period.

### Host response related to the NFkB pathway and correlations with the rumen microbiota

**Fig 1:**
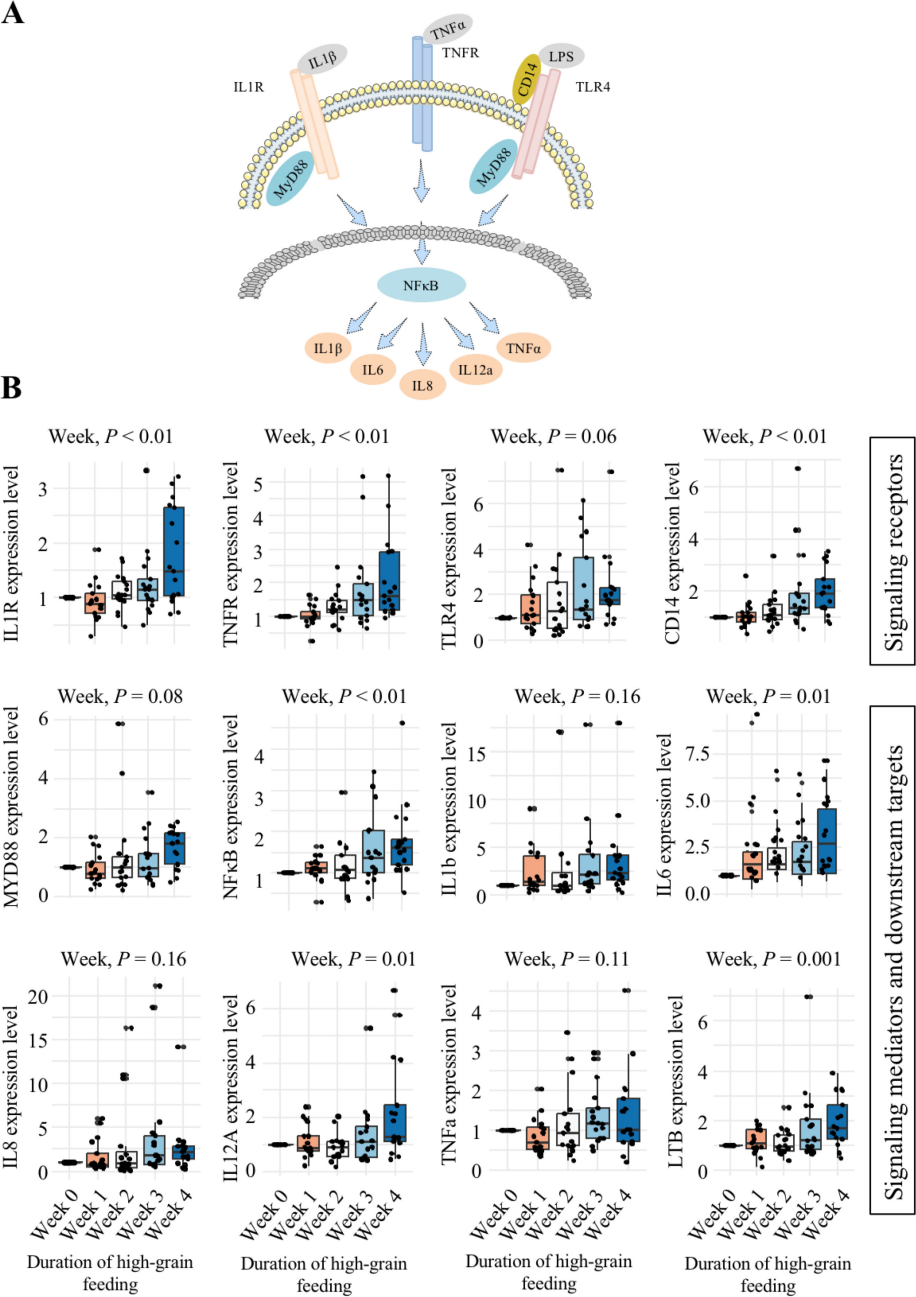
Major genes associated with the NFkB pathway (**A**) and their expression (**B**) in ruminal papillae biopsies due to the duration of high-grain feeding in non-lactating Holstein cows. Week 0 represents a week when a forage diet was offered.

Figure 1A illustrates the association of the evaluated genes within the NFkB pathway, and Fig. 1B shows the effects of duration of high-grain feeding on these genes. These results show that the expression of most genes evaluated was affected according to duration on high grain. Specifically, from week 2 on high-grain onwards, the expression of the gene interleukin 1 receptor (IL1R) increased (*P* < 0.01) compared to week 0. In addition, from week 3 onwards, the expression of genes cluster of differentiation 14 (CD14) and lymphotoxin beta (LTB) increased (*P* < 0.01) compared to week 0. Moreover, in week 4 on high grain, the expression of tumor necrosis factor receptor 2 (TNFR), NFkB, and interleukins 6 and 12A (IL6, and IL12A) increased (*P* < 0.01) compared to week 0.

Significant correlations were found between certain genes related to the NFkB pathway and the rumen microbial relative abundance (Table S2 of supplementary material). For example, the relative abundance of *Ruminococcaceae* UCG-005 was positively correlated with the genes TNFR (*P* < 0.001, r = 0.55) and CD14 (*P* < 0.001, r = 0.61). In addition, the relative abundance of a *Porphyromonadaceae* family member positively correlated with toll-like receptor 4 (TLR4) (*P* < 0.001, r = 0.58). We also found that interleukin 1b (IL1b) positively correlated with an uncultured *alpha proteobacterium* (from phylum *Proteobacteria*) (*P* < 0.001, r = 0.57) and with *Clostridiales* vadinBB60 group gut metagenome (*P* < 0.001, r = 0.57).

### Diversity of the bacterial community in the rumen and feces

#### Alpha diversity

**Fig 2:**
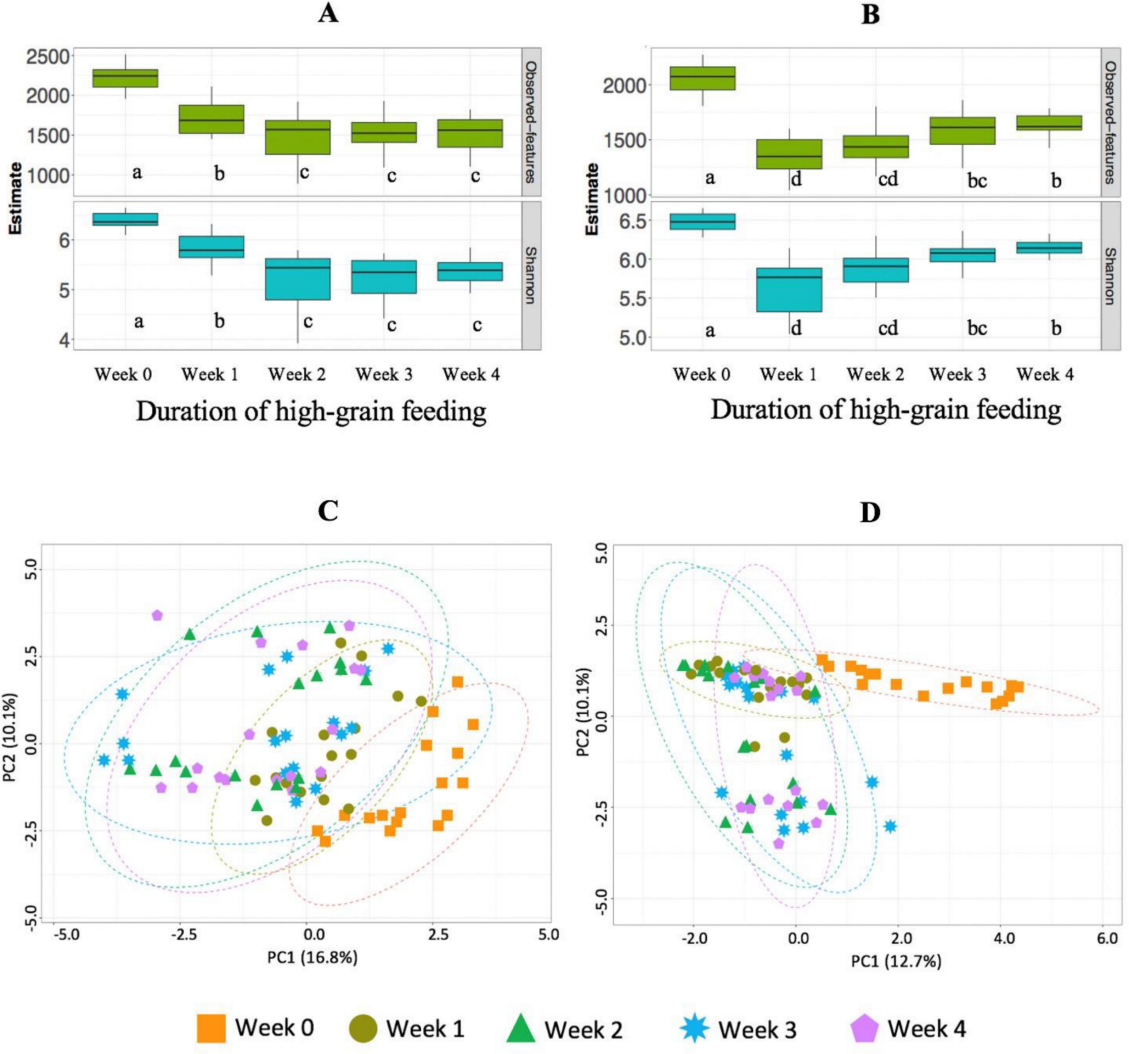
Alpha diversity of the bacterial communities in ruminal fluid (**A**) and feces (**B**), and Aitchison beta diversity plots of the bacterial communities in the ruminal fluid (**C**) and feces (**D**) for samples collected in the week of forage feeding (Week 0) and in 4 weeks of high-grain feeding (week 1 to week 4) in Holstein cows. a,b,c: significant difference across weeks (*P* < 0.01).

In the rumen, at the start of the high-grain feeding, we found a reduction in bacterial richness and diversity indices (Fig. 2A), as revealed by the number of observed features and Shannon indices (*P* < 0.05). In addition, alpha diversity seems to be further negatively affected by advanced duration on high grain (*P* < 0.05). However, estimates of richness and diversity stabilized after week 3 on high-grain.

In feces, we also observed a strong reduction (*P* < 0.01) in species richness and diversity indices immediately at the start of high-grain feeding (Fig. 2B). However, diversity estimates recovered progressively with duration on high grain, so the alpha diversity was greater (*P* < 0.01) in weeks 3 and week 4 compared to week 1 of high-grain feeding.

#### Beta diversity

In the rumen, beta diversity analysis through a principal component analysis plot showed that the bacterial communities clustered separately according to duration on high grain. There was an evident clustering of week 0 from the rest, indicating strong shifts in the bacterial profile, especially at the start of the high-grain feeding (Fig. 2C). In addition, the pairwise comparison with PERMANOVA indicated significant differences (*P* < 0.05) between week 0 and week 1, as well as between week 1 and the rest of weeks on high grain, suggesting that duration on high-grain further impacts the microbial community structure.

In feces, the bacterial communities also clustered separately according to the duration of high-grain feeding, indicating a strong overall shift in the bacterial profile, especially at the start of the high-grain feeding (Fig. 2D). The pairwise comparison with PERMANOVA indicated significant differences (*P* < 0.05) between week 1 and weeks 3, and 4.

### Bacterial taxa are mostly affected by the duration of high-grain feeding

**Fig 3:**
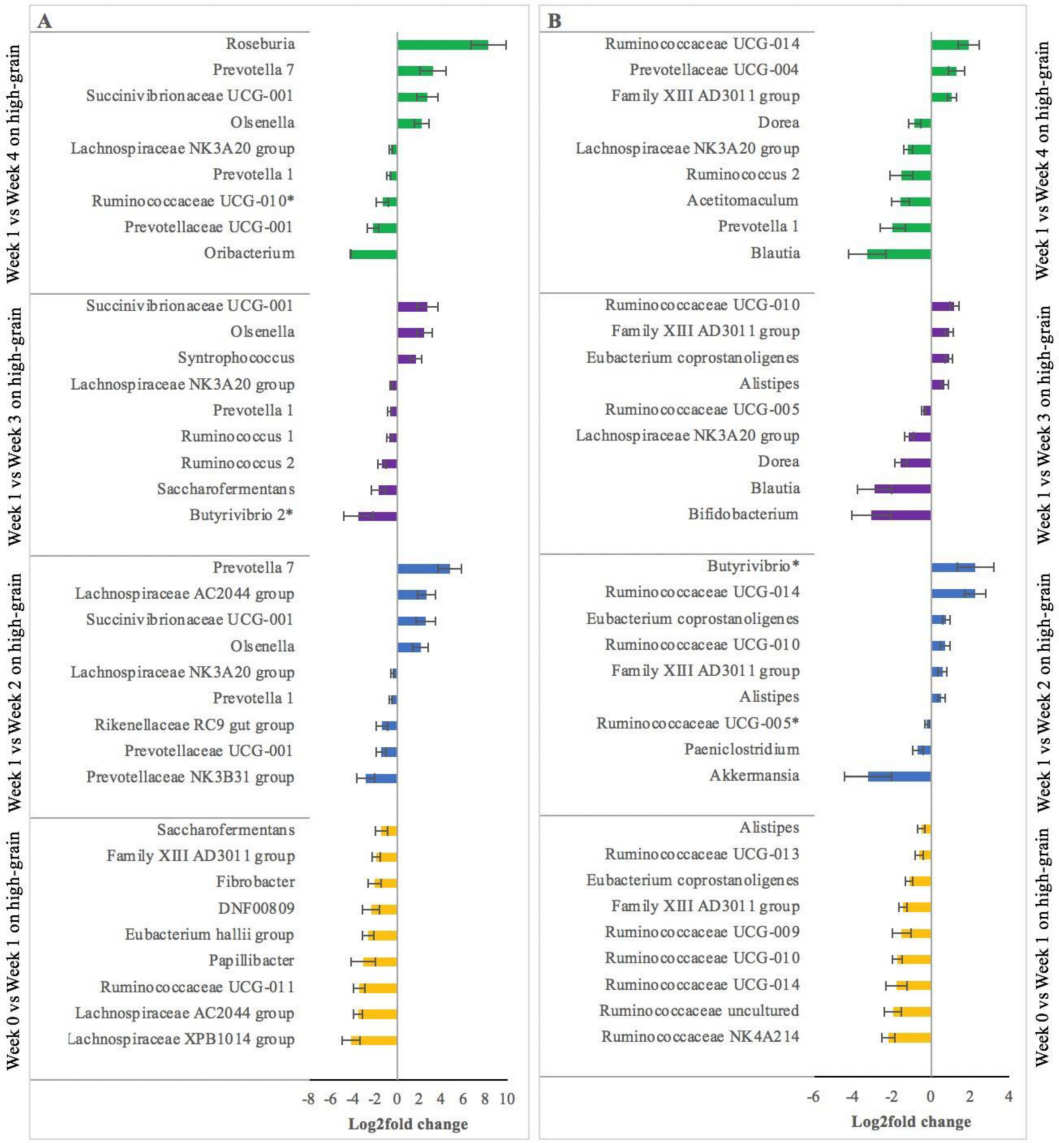
Strongest changes (*P* ≤ 0.05) or tendencies (*P > 0.05 and ≤0.10) in the relative abundance of ruminal (**A**) or fecal (**B**) bacteria of non-lactating Holstein cows when comparing forage feeding (week 0) vs week 1 on high grain (orange), week 1 vs week 2 on high grain (blue), week 1 vs week 3 on high grain (purple) and week 1 vs week 4 on high grain (green). Negative values indicate a reduction in relative abundance compared to the reference week (i.e., all bacterial taxa shown decreased in relative abundance when cows were switched from forage to week 1 on high grain.

The change from week 0 to week 1 of high-grain feeding resulted in major changes in the relative abundance of several families and genera in the rumen (Fig. 3A). For example, reductions (*P* < 0.01) were observed in genera *Saccharofermentans*, Family XIII AD3011, *Fibrobacter*, *Eubacterium Hallii*, *Papillibacter*, and the families *Ruminococcaceae* UCG-011, *Lachnospiraceae* AC2044, and *Lachnospiraceae* XPB1014. Results also showed that some genera are affected differently by duration on high grain. For example, compared to week 1, *Prevotella* 1 and *Lachnospiraceae* NK3A20 were consistently negatively affected (*P* < 0.01) from week 2 on high-grain onwards. On the other hand, the relative abundance of genera *Oribacterium* was only negatively affected in week 4 on high grain. Results further show a strong increase in taxa *Roseburia, Prevotella* 7, *Olsenella*, and *Succinivibrionaceae* UCG-001 (*P* < 0.01) in week 4 of high-grain feeding.

In feces (Fig. 3B), negative effects (*P* < 0.05) were observed at the start of high-grain feeding in the relative abundance of families *Ruminococcaceae* NK4A214, *Ruminococcaceae* UCG-014, and *Ruminococcaceae* UCG-010. Some of the bacterial families or genera that were negatively affected (*P* < 0.05) only after week 3 of high-grain feeding include *Bifidobacterium*, *Blautia*, *Ruminococcus* 1, *Prevotella* 1, and *Acetitomaculum*. Interestingly, some of the bacterial families that were negatively affected at the start of the high-grain feeding displayed an increment from week 2 onwards compared to week 1 on high grain. For example, the relative abundance of *Ruminococcaceae* UCG-014 and *Ruminococcaceae* UCG-010 increased (*P* < 0.05) from week 2 to week 4, despite the strong reduction experienced at the start of high-grain feeding.

### Ruminal metabolites and associations with microbial dynamics

**Fig 4:**
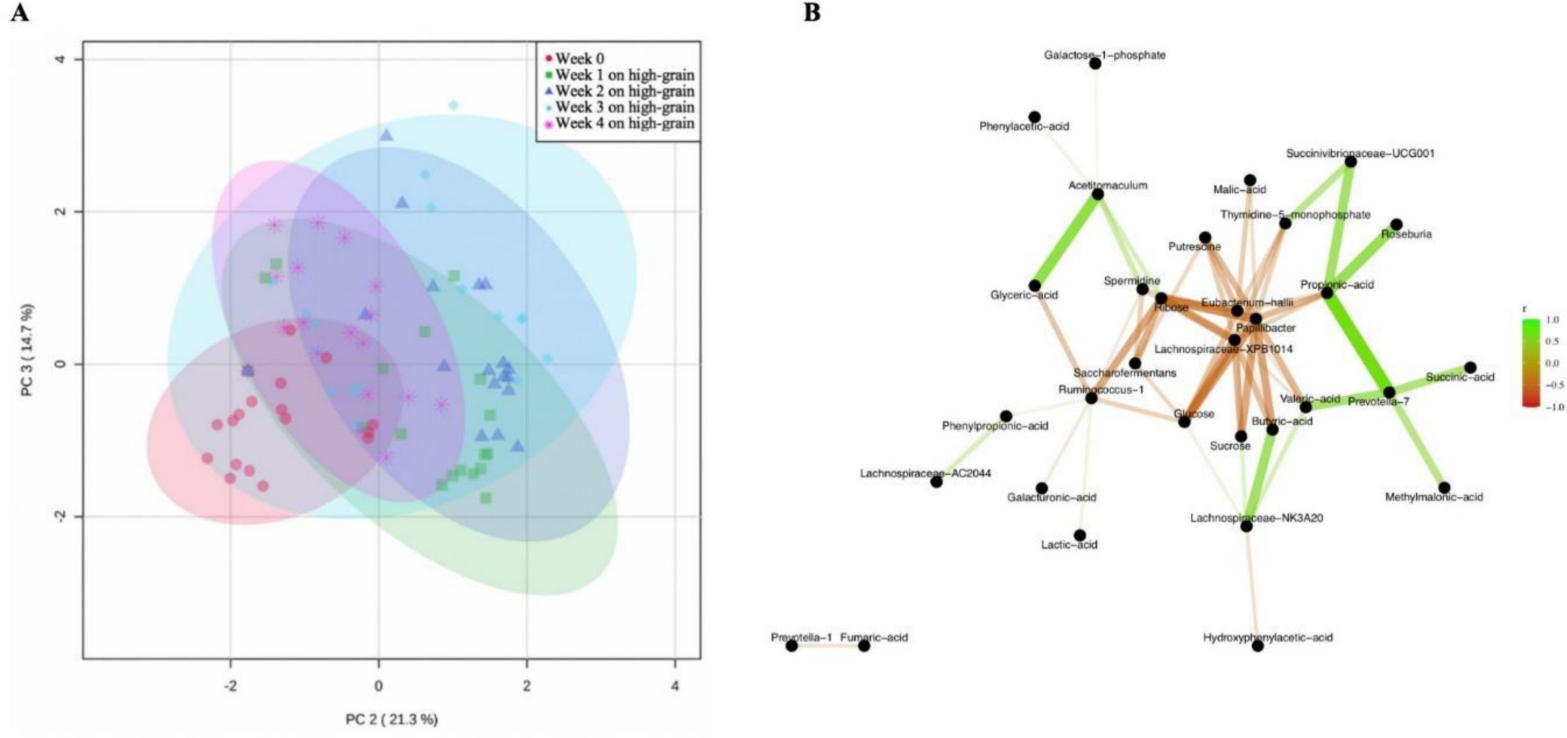
(**A**) Principal component analysis for the variation of metabolic profile in the ruminal fluid of non-lactating Holstein cows for the week of forage feeding (week 0) and in each of the 4 weeks of high-grain feeding. (**B**) Correlation network illustrating the strongest associations (r ≥ |0.40|; *P* < 0.01) found between the relative abundance of the ruminal bacterial taxa affected by duration of high-grain feeding and ruminal metabolites in non-lactating Holstein cows.

Analyses through principal components of the ruminal metabolic profile revealed a separate clustering of week 0 compared to the weeks of high-grain feeding (Fig. 4A). All ruminal metabolites measured are listed in Table S3 in the supplementary material, including 12 biogenic amines detected. In the ruminal fluid samples, the change to high-grain resulted in greater concentrations primarily on intermediates of the Embden-Meyerhof-Parnas pathway as well as the pentose phosphate pathway, two of the major routes in the metabolism of carbohydrates in ruminal bacteria. Specifically, with the change from week 0 to week 1, there were increments (*P* < 0.05) on glucose 1-phosphate, fructose 6-phosphate, succinic acid, glyceric acid, and ribose 5-phosphate. In addition, the change from week 0 to week 1 resulted in increments (*P* < 0.05) in molecules used in nucleotide synthesis, such as adenosine monophosphate and guanosine monophosphate. The concentration of phenylpropionic acid decreased due to the switch to a high-grain diet and remained low during the rest of the grain feeding. On the other hand, we found significant increments (*P* < 0.05) in the biogenic amines histamine, putrescine, spermidine, spermine, and aminovaleric acid with the change from week 0 to week 1 on high grain. The high levels of these amines were maintained throughout the high-grain regime.

Significant correlations (*P* < 0.01) were found (Fig. 4B) between the relative abundance of ruminal bacteria and several ruminal metabolites. For example, *Prevotella* 7 correlated positively with succinic acid and with propionic acid. *Eubacterium hallii* negatively correlated with ribose, sucrose, glucose, propionic acid, butyric acid, and valeric acid. *Acetitomaculum* correlated positively with ribose and glyceric acid. *Ruminococcus* correlated negatively with glucose; *Succinivibrionaceae* UCG-001 correlated positively with propionic acid.

**Fig 5:**
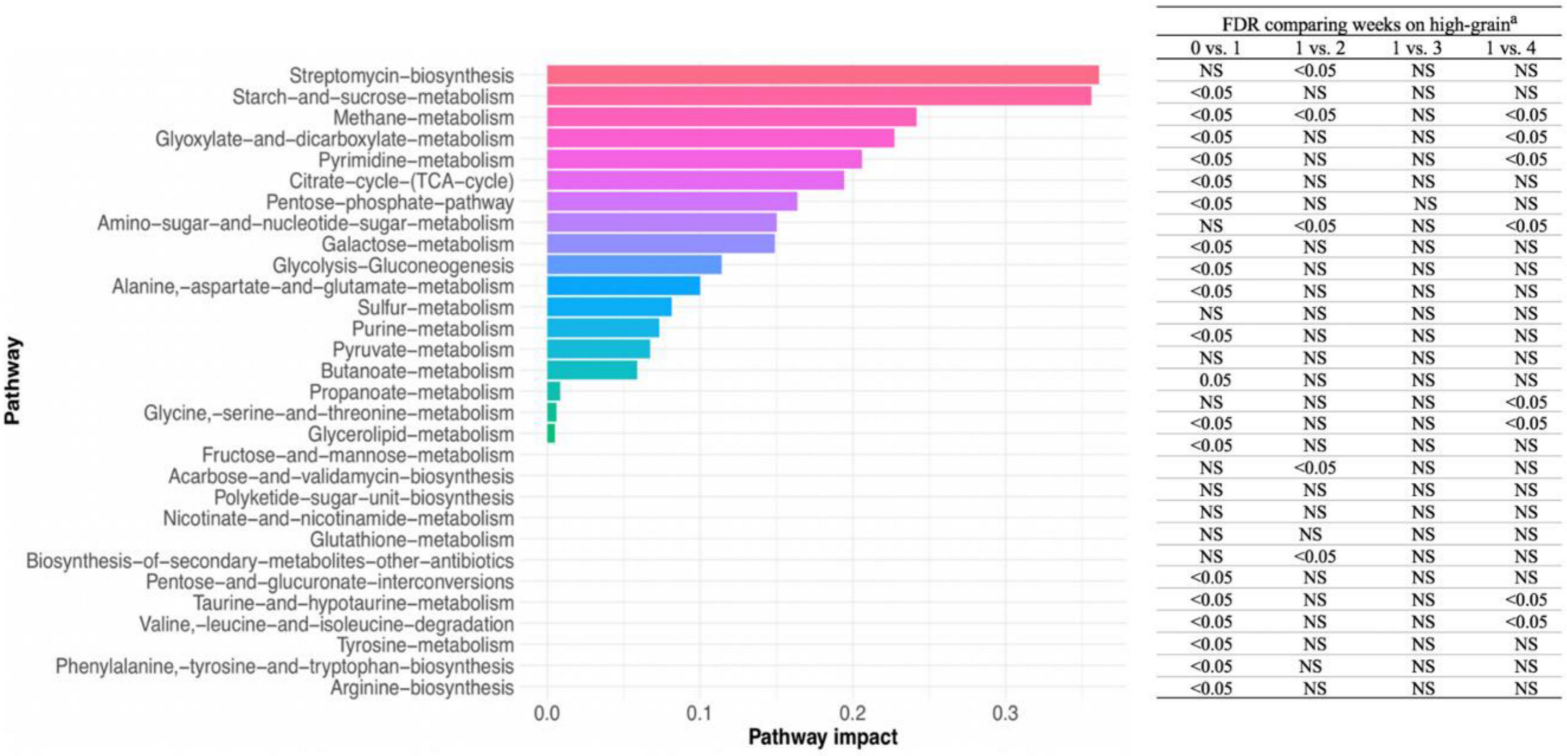
Pathways detected during the experiment ranked according to their impact, and the significance level of the pathway enrichment during the experimental weeks. ^a^: 0 vs 1: false discovery rate (FDR) for the comparison of the change from week 0 (forage diet) vs week 1 on a high-grain diet; 1 vs 2: FDR for the comparison of week 1 vs week 2 on a high-grain diet; 1 vs 3: FDR for the comparison of week 1 vs week 3 on a high-grain diet; 1 vs 4: FDR for the comparison of week 1 vs week 4 on a high-grain diet.

**Fig 6:**
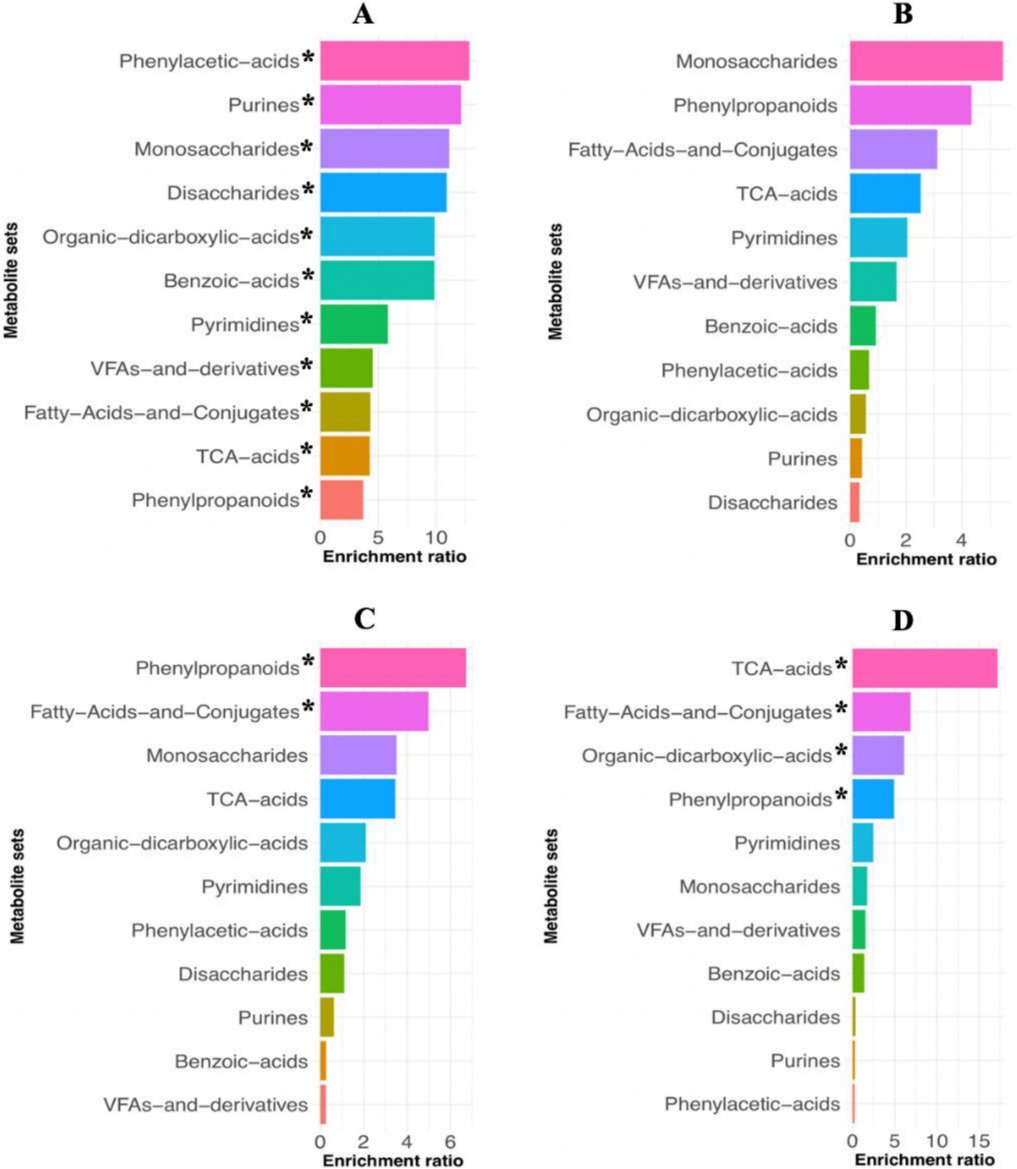
Metabolic compounds ranked according to enrichment ratio detected during the experiment. The enrichment ratio was calculated as the number of hits of a metabolic compound (indicated by the *statistic Q* column in the resulting output) divided by the expected number of hits of the corresponding compound. (**A**) Week 0 (forage diet) vs week 1 on a high-grain diet; (**B**) week 1 vs week 2 on a high-grain diet; (**C**) week 1 vs week 3 on a high-grain diet; (**D**) week 1 vs week 4 on a high-grain diet. *indicates significant enrichment with a false discovery rate of <0.05.

In agreement with the changes found in ruminal microbial metabolites, 18 major metabolic pathways were detected during the experiment, and were ranked according to their impact. These pathways were enriched differently according to duration on high grain (Fig. 5). Pathways with greater impact were streptomycin biosynthesis, starch and sucrose metabolism, methane metabolism, glyoxylate and dicarboxylate metabolism, pyrimidine metabolism, citrate cycle, pentose phosphate pathway, and amino sugar and nucleotide sugar metabolism. All of these major pathways were significantly enriched with the transition to high-grain feeding, except streptomycin metabolism, which was enriched only in week 2 on high grain along with amino sugar and nucleotide sugar metabolism. In week 3 on high grain, none of these pathways were enriched. However, in week 4 on high grain, the pathways of methane metabolism, glyoxylate and dicarboxylate metabolism, and pyrimidine metabolism were again significantly enriched. This effect also resulted in differential enrichment of microbial metabolites. For example, the change from week 0 to week 1 on high-grain resulted in enrichment primarily of phenylacetic acids, purine bases, and monosaccharides. After cows had been on high grain for 2 weeks, the top metabolites enriched were monosaccharides, phenylpropanoids, and fatty acids and conjugates. However, after 3 weeks on high grain, microbial metabolite enrichment was mainly associated with the TCA cycle, fatty acids and conjugates, and organic dicarboxylic acid (Fig. 6).

## DISCUSSION

Despite extensive research regarding the effects of high-grain diets on production performance and gut fermentation in cattle, limited quantitative data are showing how cows cope with extended duration of high-grain feeding. Elucidating this research gap should have important applications; for example, in cross-over experiments typically used in studies with dairy cattle (i.e., Latin Squares), it has commonly been assumed that a period between 2 and 3 weeks after the diet transition is sufficient for animals to adjust to the new diet. Our findings suggest that cattle need a longer time to adjust. This is particularly important to consider notably when switching from diets with different compositions, and when evaluating parameters related to the microbiome or host immune response.

Our results showed that the high-grain diet induced epithelial inflammation through the expression of genes involved in the NFkB pathway. More specifically, the genes IL6, IL12, IL1R, CD14, LTB, TNFR, and NFkB showed an increase on week 4 on high grain. The increased expression in genes associated with inflammation is in agreement with the observed increment in liver enzymes and acute phase proteins with high-grain feeding (26) and suggests an active role of local inflammation in systemic inflammation and liver tissue damage due to high-grain feeding. In this context, IL12 is closely involved in the differentiation of naive T cells into Th1 cells (27), and it stimulates the production of interferon-gamma (IFN-γ) and TNF-alpha. Therefore, supporting our findings, this gene has a crucial role in triggering a potent pro-inflammatory process in cows.

The activation of CD14 and TLR-4 leads to the activation of the signaling pathway mediated by NFkB as well as the release of IL8 (28, 29). Therefore, increased NFkB expression should coincide with increased production of pro-inflammatory cytokines. In this study, this response was observed, especially in weeks 3 and 4 of high-grain feeding, which further shows an increased inflammatory response with advanced duration on this feeding regime. The latter findings agree with results observed for LTB, an important regulator of the innate immune response (30). Furthermore, supporting our findings, the expression of IL6 has been previously demonstrated to increase in high-grain feeding (31), and the levels measured in our study indicated epithelial inflammation.

Expression of IL1 and TNF-alpha shows a synergistic activity (32). In the present study, the receptors of both cytokines increased, particularly from week 3 on high grain. This suggests that negative impacts may aggravate with duration of this diet. In this context, the lack of an effect of high grain on some of the genes involved in the NFkB pathway may be due to developed tolerance. For example, an *in vitro* study exposing ruminal epithelial cells to bacterial toxins revealed tolerance toward this endotoxin (33), which agrees with our findings showing no effect of high grain on TNF-alpha and a reduction of TLR4 after reaching a maximum level in week 3.

The correlations found between genes associated with the NFkB pathway and certain rumen microbial taxa suggest possible modulation of host immune response by the rumen microbiome. For example, proinflammatory molecules such as LPS are known to be released during ruminal bacterial growth and can trigger the host immune response (23, 34). However, also Gram-positive bacteria have been associated with an activation of the immune response in cows fed high-grain diets (22). In this regard, studies in other species demonstrated that the cell wall of Gram-positive bacteria can trigger the production of TNF-alpha, which signals through its TNFR, regulating the cascade of events inducing the inflammatory process (35–37). Thus, the positive correlation found between *Ruminococcaceae* UCG-005 and TNFR as well as with CD14 may be due to stimulation of these genes by the proliferation of *Ruminococcaceae* UCG-005, leading to an inflammatory response with increased duration of high-grain feeding. Similarly, LPS is also known to be highly produced by *Proteobacteria* (38); thus, the proliferation of *alpha proteobacterium* may have contributed to the increased endotoxin release, leading to increased expression of IL1b as part of the host proinflammatory response.

Contrary to what is commonly believed, our results show that the activation of the NFkB pathway during high-grain feeding could be due not only to LPS shedding but also to the proliferation and growth of Gram-positive bacteria. Furthermore, the pathway was activated throughout the 4 weeks of high-grain feeding in our experiment, suggesting that the host immune system was still reacting to shifts in microbial composition.

Our observations also revealed changes in the gut microbiome throughout the 4 weeks after the diet switch. For example, a decline in rumen bacterial alpha diversity was found at the start of high-grain feeding but diversity indices stabilized after 3 weeks on high grain. The increments found on the propionate-producing taxa *Prevotella* and *Succinivibrionaceae* suggest tolerance to low ruminal pH, which decreased around 0.44 units with high grain. This finding agrees with the correlations found between propionic acid (and its intermediate, succinic acid) and the taxa *Prevotella* and *Succinivibrionaceae* resulting in the enrichment of TCA acids and organic dicarboxylic acids suggesting greater nutrient metabolization with advanced duration on high grain.

Another interesting finding was that, after the initial decline observed immediately after the diet switch, the alpha diversity of the fecal bacterial community stabilized with advanced duration of high-grain feeding. This stabilization may be due to the recovery of key bacterial taxa, such as *Ruminococcaceae* UCG-014. Thus, suggesting adaptation of these taxa to tolerate low hindgut pH. The adaptation of some of these taxa to low pH may reflect the activation of adaptive mechanisms to prevent negative effects of low pH, such as the employment of enzyme-catalyzed reactions that consume protons, deployment of reactions that produce basic compounds to help neutralize the low pH, and elimination of protons from the bacterial cells at the expense of ATP consumption (39). This may be an indication of microbial stability and capacity to recover after a perturbation event (40). Thus, our findings suggest a steadier microbiota in the hindgut compared to the rumen, and therefore increased resistance to acidogenic diets in the lower segment of the gastrointestinal tract of cattle.

The changes found in the ruminal bacterial community were in agreement with variations observed in the metabolic profile. For example, the increment in intermediates of the EMP glycolytic pathway as well as the pentose phosphate route reflect the proliferation of starch-digesting bacterial taxa. In addition, the generation of the nucleotide bases such as thymidine 5 monophosphate and their strong correlation with starch fermenting bacteria may reflect the use of these metabolites to support bacterial growth and bacterial protein synthesis, which usually increases with greater availability of dietary nitrogen and starch. Concomitant to the increase in starch degradation, there was an increment in the generation of biogenic amines. The generation of most biogenic amines was particularly greater immediately with the change from week 0 to week 1. This may reflect greater amino acid decarboxylation in the rumen due to the increased availability of dietary protein. In this regard, some of the detected biogenic amines have been suggested to have pro-inflammatory activity, suggesting an increased risk of systemic inflammation with duration of high-grain feeding. These findings support previous reports showing an increase in acute-phase proteins during high-grain feeding (26). In this context, because butyric acid is known to have anti-inflammatory effects (41), the positive correlation between *Lachnospiraceae* NK3A20 and butyric acid suggests that promoting the growth of this taxon may contribute to cattle health when fed high-grain diets.

The metabolic pathways detected in this study agreed with the enrichment of compounds due to the duration of the high-grain diet. It is interesting to notice how the metabolites enrichment analysis reflected the adaptive changes of microbial metabolism: while in the first weeks on a high-grain simple carbohydrates resulted more enriched, their ranking positions were gradually substituted by products of their fermentation. At the same time, the lower activity of the fibrolytic bacteria was reflected by the enrichment of phenylpropanoids (42). Furthermore, the enrichment primarily of phenylacetic acids, purines, and monosaccharides observed due to the change from forage to a high-grain diet may reflect increased bacterial growth and substrate metabolism.

Purines and monosaccharides are used for bacterial growth (43). Thus, shifts in the enrichment of these compounds with duration on high-grain may reflect changes in the rumen microbial profile and biomass. For example, after 3 weeks on high-grain, utilization of starch by amylolytic bacteria generates propionate and intermediates such as fumarate and succinate (classified as intermediates of the TCA cycle, significantly enriched in week 4 on high grain). Moreover, during microbial protein production, intermediary molecules including oxaloacetate, fumarate, alpha-ketoglutarate, and citrate are produced (43). On the other hand, the high impact of the methane metabolism pathway in the rumen may reflect the intense crosstalk among ruminal microbial communities during methanogenesis. For example, large amounts of CO_2_ and H_2_ are generated by bacteria and protozoa and subsequently utilized by archaea (44).

Surprisingly, results show that predicted streptomycin synthesis was the most impactful pathway in our study. Streptomycin is produced by *Streptomyces* from D-glucose to compete with other bacteria (45). However, in our study, this genus did not show increased relative abundance due to diet and was detected only in a few samples. Enrichment of this pathway has been reported in cattle due to carbohydrate or amino sugar and nucleotide sugar metabolism (46, 47), which were also enriched in our study. Thus, the high impact of this pathway likely reflected the variations in carbohydrate concentrations.

### Conclusion

Our study reveals that the transition to a high-grain diet initiates a significant reduction in rumen microbial alpha diversity, which subsequently stabilizes by the third week on high-grain. Despite this initial stabilization, the high-grain feeding period is characterized by persistent alterations in microbial relative abundance, metabolic pathway activities, and the expression patterns of signaling receptors, mediators, and targets associated with the NFκB pathway. The strong proinflammatory response of cows seems to be associated with the relative abundance and proliferation of certain rumen bacterial taxa, possibly due to both Gram-positive and Gram-negative bacteria. Notably, as the high-grain feeding progresses, there is a recovery in the relative abundance of certain hindgut bacteria and an improvement in hindgut alpha diversity indices, suggesting a microbial adaptation to the lower pH environment induced by the high-grain diet. Despite a potential adaptation of the lower gut to the high-grain diet, the ruminal environment showed signs of stabilization but not complete recovery. These findings underscore the critical adaptation period of at least 4 weeks required by cows after the transition to high-grain feed. Our findings provide pivotal insights for designing and interpreting experiments that involve dietary modifications in cattle, highlighting the need for adequate adjustment periods to ensure the stability and reliability of research outcomes.

## MATERIALS AND METHODS

### Animals, experimental design, and animal management

The protocols followed in this experiment were approved by the Ethics and Animal Welfare Committee of the University of Veterinary Medicine, Vienna in accordance with the University’s Guidelines for Good Scientific Practice and authorized by the Austrian Federal Ministry of Education, Science, and Research in accordance with current legislation (protocol number: BMBWF- 68.205/0003-V/3b/2019).

Nine ruminally cannulated (Bar Diamond, Parma, ID), non-lactating, multiparous Holstein cows (916 ± 22.9 kg BW; with an average age of 11.0 ± 2.1 years) were used. Cows were housed in a stall equipped with deep litter cubicles. Water and feed were available for *ad libitum* consumption. The experiment consisted of two periods. There was an interval of 2 months between the experimental periods. The feed intake for each of the cows averaged by week is listed in Table S4 of the supplementary material.

### Diet composition, feeding and monitoring of ruminal pH

In each of the two experimental periods, cows were first fed only forage for a week (*week 0*); then, they were gradually transitioned over a week to a 65% grain diet (Table S5 in the supplementary material), which they consumed for four additional weeks (*week 1* to *week 4*). As opposed to transition and early lactation cows, the timeframe used for diet adaptation in this study was shorter, and no close-up diet was used. The main rationale behind our experimental setup was to induce gastrointestinal acidification and to accentuate the effects of the high-grain feeding using dry cows as the experimental model. Before the initiation of the study and between the two periods, cows grazed on pasture. The rations were prepared daily at 0600 using an automated feeding system (Trioliet Triomatic T15, Oldenzaal, The Netherlands) and were offered in individual feed bins, which were equipped with electronic weighing scales. Thus, feed intake was recorded for each cow daily. Ruminal pH was monitored with indwelling systems (48). Measurements were collected continuously every 15 minutes. At the end of the study, millivolt values were converted to actual pH, and data were summarized.

Samples of feed ingredients as well as diets were collected weekly. Analyses for chemical composition included ash by combustion overnight at 580°C (method 8.1); crude protein following the Kjeldahl protocol (method 4.1.1) (49); ether extracts (EE) using the Soxhlet extraction system (method 5.1.2; Extraction System B-811, Büchi, Flawil, Switzerland); NDF (method 6.5.1) and ADF (method 6.5.2) following the official analytical methods (49) using the Fiber Therm FT 12 (Gerhardt GmbH & Co. KG, Königswinter, Germany). Starch was measured with the K-TSTA kit (Megazyme Ltd., Ireland). Non-fiber carbohydrates were calculated as 100 − (% crude protein + % NDF + % ether extract + % ash); residual organic matter (ROM) was calculated by portioning NFC into starch and ROM (50). The particle size distribution of the diet was measured using the method described earlier (51).

### Collection of ruminal papillae samples and extraction of total RNA

Ruminal papillae samples were collected weekly within each experimental period. To do so, the rumen was partially emptied; the ruminal digesta contents were placed in pre-warmed insulated plastic containers. Then, the lateral ruminal wall (approximately 20 cm below the ruminal fistula) was exteriorized and washed with PBS. The papillae were sampled by cutting near the base with sterile scissors. During the forage feeding, the papillae samples were collected after 1 week of consuming the diet to allow proper adaptation; during the high-grain feeding, these samples were collected on day 2 of each week (Tuesday), 4 hours after the morning feeding. To keep consistency in the methodology, these samples were collected from the same ruminal area from each cow. Then, the collected papillae samples were immediately snap-frozen in liquid nitrogen and were transferred into 2 mL cryotubes. At the end of samplings, the rumen digesta was returned in the rumen, and papillae samples were stored at −80°C until later analysis at the end of the experiment. All equipment used were either single-use (thus discarded) or disinfected with ethanol after each sampling.

Total RNA was isolated from the ruminal papillae samples using the RNeasy Mini Qiacube Kit (Qiagen, Hilden, Germany) according to the manufacturer’s instructions, with minor changes (52). To do so, from the collected ruminal papillae samples, an aliquot of 30 mg was used by placing the aliquot directly in the lysis buffer for thawing. This aliquot was then disrupted and homogenized in 300 µL lysis buffer with ceramic beads. The RNA was eluted using 50 µL of RNase-free water. RNA quantity and quality were assessed on the Qubit Fluorometer 4.0 (Life Technologies Corporation, CA, USA) using the Qubit RNA HS Assay Kit and the Qubit RNA IQ Assay Kit (Thermo Fisher Scientific, Vienna, Austria). Samples were frozen and stored at −80°C. The average integrity number of the obtained RNA was 9.41, with a range from 8.9 to 9.9.

### Reverse transcription and gene expression analysis

We focused on several key genes associated with the NFkB pathway and its important downstream targets; namely, IL1b and its receptor (IL1R), IL6, IL8, IL12, and TNF-alpha and its receptor (TNFR). In addition, the expression of upstream NFkB genes was evaluated; namely, CD14, TLR4, LBT, and myeloid differentiation factor 88 (MYD88). A total of 2 µg of RNA per sample was used to generate single-stranded cDNA on a thermocycler (Nexus Thermocycler, Eppendorf, Vienna, Austria). The master mix for cDNA synthesis contained 4 µL of 10 × buffer reverse transcriptase, 1.6 µL of 25 × dNTP mix (100 mM), 4 µL of 10 × reverse transcription primers, 1 µL of RNAse inhibitor, 2 µL of Multiscribe reverse transcriptase, 7.4 µL of nanopure water, and 20 µL of template, in a total volume of 40 µL. Samples without reverse transcriptase nor RNAse inhibitor were used as negative controls to confirm the absence of contaminations. However, the master mix of the negative controls contained 2 µL of 10 × buffer reverse transcriptase, 0.8 of 25 × dNTP mix (100 mM), 2 µL 10 × reverse transcription primers, 5.2 µL of nanopure water, and 10 µL of template containing 1 µg RNA, in a total volume of 20 µL.

The oligo primers used for the evaluated genes are listed in Table S6 in the supplementary material. Some of these genes required the design of new primers (IL1R, TNF-alpha, TNFR, IL8, IL12), which was performed with *Primer3* and *Ensembl* database. Each sample was subject to quantitative PCR, which was performed in 10 µL volume, with 2 µL of cDNA and 8 µL of master mix. Each reaction of the master mix included 5 µL of Blue S′Green (Blue S′Green qPCR Mix Separate ROX, Biozym, Austria), 0.8 µL of forward and reverse primer, and 2.2 µL of nanopure water. Thus, the reaction contained 20 ng of cDNA template and 80 nM of primers. Each sample was run in duplicate in separate wells of the 384-well real-time PCR plate (Sarstedt, Austria). Each qPCR assay included four samples with no DNA as non-template controls. Then, RT-qPCR was performed with a qTOWER^3^ 84 real-time PCR instrument (Analytik Jena, Germany) to determine the relative copy numbers of different mRNAs. The amplifications were performed with the following protocols: stage 1: 95°C for 3 min, followed by stage 2: 40 cycles composed of 5 s at 95°C and 30 s at the estimated annealing temperature, and stage 3: melt curve analysis. The genes hypoxanthine phosphoribosyltransferase 1 (HPRT1), ornithine decarboxylase antizyme 1 (OAZ1), and 14-3-3 protein zeta/delta (YWHAZ) were used as reference genes for the normalization of mRNA content in each sample. Differences in target gene expression profiles over weeks 0, 1, 2, 3, and 4 of high-grain feeding within each experimental period were analyzed as fold change using the 2−ΔΔCt method. Briefly, for each cow, week, and target gene, the mean of the cycle threshold (Ct) of the target gene was calculated and then subtracted from the geometric mean of the Cts of the reference genes. Then, the calculated ΔCt values of the target genes were subtracted from the mean of the ΔCt values of the cows of week 0 to estimate the ΔΔCt values over the weeks 1, 2, 3, and 4. The relative expression of the target genes was then calculated using the information of 2−ΔΔCt for each experimental period.

### Collection of ruminal fluid and fecal samples

Samples of the ruminal fluid associated with solid feed particles were also collected weekly 4 h post-feeding, this sampling was performed similarly to our previously used approach (12). Briefly, samples of rumen contents were collected from four different regions (caudal ventral sac, cranial ventral sac, and two samples from the feed mat in the middle and dorsal rumen) using a disposable palpation sleeve for each collection. Ruminal contents were composited in a sterilized container and were strained through four layers of gauze. Then, 2 mL of the collected ruminal fluid was immediately snap-frozen in liquid nitrogen. Samples were stored at −80°C until later analyses for microbial communities and metabolic profiles.

Grab fecal samples were collected rectally using a palpation sleeve for each collection (19). Likewise, these samples were taken on a weekly basis 4 h post-feeding (same days and time as ruminal fluid samplings). Around 2 mL of the collected feces was placed in cryotubes using a spatula previously sterilized with 70% ethanol, and immediately snap-frozen in liquid nitrogen. Then, samples were stored at −80°C.

### DNA extraction and sequencing for ruminal and fecal samples, and amplicon processing

DNA was isolated and purified using PowerSoilPro Kit (Qiagen) through a bead-beating step at first. Briefly, for ruminal fluid, DNA extraction was conducted from 800 µL of each sample; whereas for feces, 265 mg of sample were used. The samples were treated with 100 µL of 100 mg/mL lysozyme and 10 µL of 2.5 U/mL mutanolysin as well as proteinase K. The DNA was eluted with 75 µL of C6 buffer and the concentration was measured on a Qubit 4.0 Fluorometer (Life Technologies Corporation, CA USA) using the Qubit ds DNA Assay Kit. Then, 40 µL samples of DNA were shipped to an external laboratory (Novogene, Cambridge, United Kingdom) for library preparation and sequencing.

The region V3–V4 of the 16S rRNA gene from the microbial communities was amplified with primers 341F (CCTAYGGGRBGCASCAG) and 806R (GGACTACNNGGGTATCTAAT) (53, 54). Equimolar pools of samples were sequenced using a 250 × 2 bp paired-end reads protocol for the Illumina MiSeq platform (Novogene). Multiplexed libraries were constructed by ligating sequencing adapters and indices onto purified PCR products using the Nextera XT Sample Preparation Kit. Primers were trimmed and the overlapping paired-end reads were merged.

The analysis of data for high-throughput sequencing was performed using QIIME2 v2020.2 (55) and R v4.0.0 (56). Sequence analysis initiated with demultiplexed fastq sequence files. The forward and reverse primer sequences as well as barcodes were removed from the demultiplexed sequences using the QIIME2 cut-adapt plug-in (57). Forward and reverse reads were joined using FLASH (58). Singletons and ASVs with less than 10 reads were discarded, reads were trimmed to 387 nucleotides, quality filtered, and chimeras were removed; denoising was performed with deblur (59). A minimum threshold for Phred score of Q = 20 was used for quality filtering (60, 61). Sequence taxonomy was assigned using a trained classifier for the specific 16S rRNA 341F/806R region using the last version of the SILVA database (v. 138; https://www.arb-silva.de/; (accessed 20 May 2023). QIIME2 artifacts were loaded into R using the qiime2R v0.99.23 and phyloseq v1.32.0 (62) packages.

Analysis of alpha diversity including Chao1, observed ASVs, Shannon, and Simpson indices was performed with R (version 1.4.1106). Principal coordinate analysis (PCoA) plots for beta diversity based on Aitchison distance matrix analysis were generated and visualized in two-dimensional plots. Good’s coverage test was performed to evaluate whether adequate sampling depth was achieved. Furthermore, taxa from phylum to genus were summarized.

### Evaluation of the rumen fluid metabolome

A portion of the ruminal fluid collected was analyzed for metabolic profile. This analysis was performed by anion exchange chromatography coupled to high-resolution mass spectrometry (IC-HR-MS on a Dionex Integrion HPIC system coupled to a Q Exactive Orbitrap mass spectrometer, both from Thermo Scientific). To do so, a 20 µL aliquot of thawed sample was shaken with 980 µL of acetonitrile/water (80/20, vol/vol) at 4°C for 10 min, centrifugation was performed at 14,350 *× g* for 10 min, and the supernatant was diluted 10-fold with acetonitrile/water (20/80, vol/v) prior to IC-HR-MS measurement.

In addition, biogenic amines (alpha-aminobutyric acid, aminovaleric acid, beta-alanine, cadaverine, ethanolamine, gamma-aminobutyric-acid, histamine, putrescine, phenylethylamine, pyrrolidine, spermidine, spermine) were measured by LC-MS/MS using a modified protocol based on Biocrates’ MxP Quant 500 kit (Innsbruck, Austria). To do so, 10 µL of rumen fluid sample or different volumes of calibration stock solutions containing between 0.009 and 9 mg/L of all analytes and 30 µL of internal standard solution containing 10 mg/L ^13^C-putrescine in acetonitrile/water (50/50, vol/vol) were placed in a 96-well plate and evaporated under a stream of nitrogen. Subsequently, 50 µL of derivatization reagent (ethanol/water/pyridine/PITC 31.7/31.7/31.7/5.0, vol/vol/vol/vol) was added and the plate was covered, shaken for 20 s, and placed in the dark at room temperature for derivatization of amines. After 1 h of derivatization, the derivatization reagent was evaporated under nitrogen. Then, analytes were extracted by shaking in 300 µL of methanol containing 4.9 mM ammonium acetate for 30 min. Subsequently, the extracts were centrifuged, and measurements were performed as previously outlined (63).

### Statistical analyses

Microbial data were analyzed separately within each sample type (rumen or feces). Alpha diversity was compared through boxplots constructed with R using the package ggplot2 (64); comparisons were made using a *t*-test and the *P*-values were adjusted with the Benjamini-Hochberg method. An overall comparison of the community composition was first performed through permutational multivariate analyses of variance (PERMANOVA) in R and using the vegan package (Adonis function) (65), where the week of feeding was used as the main effect. Differential relative abundance at the taxonomic levels of phylum, family, and genus were analyzed in R using Maaslin2 (66). The statistical model included the fixed effect of week of high-grain feeding. The adjustment of the *P*-values for all microbial data including the pairwise comparisons was performed with the Benjamini-Hochbergh method for false discovery rate.

The ruminal metabolome data were assessed with MetaboAnalyst (v. 5.0) to evaluate the effect of the duration of high-grain feeding (67). In addition, pathway analysis and metabolite set enrichment analysis according to the duration of high-grain diet were performed. Furthermore, correlation analyses between the ruminal metabolome and the ruminal bacterial community were performed first using the Spearman rank correlation to identify the strongest associations with SAS. Once such associations were identified, a correlation network was generated with R using the igraph package.

Gene expression of ruminal papillae was analyzed with SAS, and cow within block was included in the statistical model as a random effect; data from different weeks from the same cow in the same treatment were processed as repeated measures. The PDIFF option was included, which allowed multiple comparisons of means throughout the evaluated time points. Then, boxplots were constructed using the packages ggplot2 with R (64). We also evaluated correlations between the expression of genes associated with the NFkB pathway and the relative abundance of the ruminal microbiome using Spearman correlation analysis with SAS. Statistical significance was declared when *P* ≤ 0.05 and tendency is indicated if *P* > 0.05 and ≤0.10.

## Data Availability

Sequencing data were deposited on the National Center for Biotechnology Information (NCBI) database, with the BioProject accession number PRJNA909964 for fecal samples and PRJNA909617 for ruminal fluid samples.

## SUPPLEMENTAL MATERIAL

Supplemental tables spectrum.00944-24-s0001.docx Tables S1-S6.

ASM does not own the copyrights to Supplemental Material that may be linked to, or accessed through, an article. The authors have granted ASM a non-exclusive, world-wide license to publish the Supplemental Material files. Please contact the corresponding author directly for reuse.

## References

[1] Fernando SC, Purvis HT, Najar FZ, Sukharnikov LO, Krehbiel CR, Nagaraja TG, Roe BA, Desilva U. 2010. Rumen microbial population dynamics during adaptation to a high-grain diet. Appl Environ Microbiol 76:7482–7490. doi:10.1128/AEM.00388-10 20851965 PMC2976194

[2] Goad DW, Goad CL, Nagaraja TG. 1998. Ruminal microbial and fermentative changes associated with experimentally induced subacute acidosis in steers. J Anim Sci 76:234–241. doi:10.2527/1998.761234x 9464904

[3] Steele MA, Vandervoort G, AlZahal O, Hook SE, Matthews JC, McBride BW. 2011. Rumen epithelial adaptation to high-grain diets involves the coordinated regulation of genes involved in cholesterol homeostasis. Physiol Genomics 43:308–316. doi:10.1152/physiolgenomics.00117.2010 21245418

[4] Bevans DW, Beauchemin KA, Schwartzkopf-Genswein KS, McKinnon JJ, McAllister TA. 2005. Effect of rapid or gradual grain adaptation on subacute acidosis and feed intake by feedlot cattle. J Anim Sci 83:1116–1132. doi:10.2527/2005.8351116x 15827257

[5] Brown MS, Ponce CH, Pulikanti R. 2006. Adaptation of beef cattle to high-concentrate diets: performance and ruminal metabolism. J Anim Sci 84:E25–E33. doi:10.2527/2006.8413_supplE25x 16582090

[6] Chibisa GE, Mutsvangwa T. 2013. Effects of feeding wheat or corn-wheat dried distillers grains with solubles in low- or high-crude protein diets on ruminal function, omasal nutrient flows, urea-N recycling, and performance in cows. J Dairy Sci 96:6550–6563. doi:10.3168/jds.2013-6622 23932131

[7] Ranathunga SD, Kalscheur KF, Anderson JL, Herrick KJ. 2018. Production of dairy cows fed distillers dried grains with solubles in low- and high-forage diets. J Dairy Sci 101:10886–10898. doi:10.3168/jds.2017-14258 30292550

[8] Nasrollahi SM, Zali A, Ghorbani GR, Khani M, Maktabi H, Beauchemin KA. 2019. Effects of increasing diet fermentability on intake, digestion, rumen fermentation, blood metabolites and milk production of heat-stressed dairy cows. Animal 13:2527–2535. doi:10.1017/S1751731119001113 31115287

[9] Kairenius P, Leskinen H, Toivonen V, Muetzel S, Ahvenjärvi S, Vanhatalo A, Huhtanen P, Wallace RJ, Shingfield KJ. 2018. Effect of dietary fish oil supplements alone or in combination with sunflower and linseed oil on ruminal lipid metabolism and bacterial populations in lactating cows. J Dairy Sci 101:3021–3035. doi:10.3168/jds.2017-13776 29428753

[10] McAllister TA, Bae HD, Jones GA, Cheng KJ. 1994. Microbial attachment and feed digestion in the rumen. J Anim Sci 72:3004–3018. doi:10.2527/1994.72113004x 7730196

[11] Bergman EN. 1990. Energy contributions of volatile fatty acids from the gastrointestinal tract in various species. Physiol Rev 70:567–590. doi:10.1152/physrev.1990.70.2.567 2181501

[12] Castillo-Lopez E, Ramirez Ramirez HA, Klopfenstein TJ, Hostetler D, Karges K, Fernando SC, Kononoff PJ. 2014. Ration formulations containing reduced-fat dried distillers grains with solubles and their effect on lactation performance, rumen fermentation, and intestinal flow of microbial nitrogen in Holstein cows. J Dairy Sci 97:1578–1593. doi:10.3168/jds.2013-6865 24440246

[13] Jenkins TC, Wallace RJ, Moate PJ, Mosley EE. 2008. Board-invited review: recent advances in biohydrogenation of unsaturated fatty acids within the rumen microbial ecosystem. J Anim Sci 86:397–412. doi:10.2527/jas.2007-0588 18042812

[14] Na SW, Guan LL. 2022. Understanding the role of rumen epithelial host-microbe interactions in cattle feed efficiency. Anim Nutr 10:41–53. doi:10.1016/j.aninu.2022.04.002 35647325 PMC9117530

[15] Wu HJ, Wu E. 2012. The role of gut microbiota in immune homeostasis and autoimmunity. Gut Microbes 3:4–14. doi:10.4161/gmic.19320 22356853 PMC3337124

[16] Raabis S, Li W, Cersosimo L. 2019. Effects and immune responses of probiotic treatment in ruminants. Vet Immunol Immunopathol 208:58–66. doi:10.1016/j.vetimm.2018.12.006 30712793 PMC6526955

[17] Mao S, Zhang M, Liu J, Zhu W. 2015. Characterising the bacterial microbiota across the gastrointestinal tracts of dairy cattle: membership and potential function. Sci Rep 5:16116. doi:10.1038/srep16116 26527325 PMC4630781

[18] Mao SY, Zhang RY, Wang DS, Zhu WY. 2013. Impact of subacute ruminal acidosis (SARA) adaptation on rumen microbiota in dairy cattle using pyrosequencing. Anaerobe 24:12–19. doi:10.1016/j.anaerobe.2013.08.003 23994204

[19] Plaizier JC, Li S, Danscher AM, Derakshani H, Andersen PH, Khafipour E. 2017. Changes in microbiota in rumen digesta and feces due to a grain-based subacute ruminal acidosis (SARA) challenge. Microb Ecol 74:485–495. doi:10.1007/s00248-017-0940-z 28175972

[20] Zhang R, Zhu W, Mao S. 2016. High-concentrate feeding upregulates the expression of inflammation-related genes in the ruminal epithelium of dairy cattle. J Anim Sci Biotechnol 7:42. doi:10.1186/s40104-016-0100-1 27478614 PMC4966727

[21] Guo J, Chang G, Zhang K, Xu L, Jin D, Bilal MS, Shen X. 2017. Rumen-derived lipopolysaccharide provoked inflammatory injury in the liver of dairy cows fed a high-concentrate diet. Oncotarget 8:46769–46780. doi:10.18632/oncotarget.18151 28596485 PMC5564522

[22] Ricci S, Pacífico C, Kreuzer-Redmer S, Castillo-Lopez E, Rivera-Chacon R, Sener-Aydemir A, Rossi G, Galosi L, Biagini L, Schwartz-Zimmermann HE, Berthiller F, Reisinger N, Petri RM, Zebeli Q. 2024. Integrated microbiota-host-metabolome approaches reveal adaptive ruminal changes to prolonged high-grain feeding and phytogenic supplementation in cattle. FEMS Microbiol Ecol 100:fiae006. doi:10.1093/femsec/fiae006 38281064 PMC10858391

[23] Zhao C, Liu G, Li X, Guan Y, Wang Y, Yuan X, Sun G, Wang Z, Li X. 2018. Inflammatory mechanism of rumenitis in dairy cows with subacute ruminal acidosis. BMC Vet Res 14:135. doi:10.1186/s12917-018-1463-7 29673406 PMC5909223

[24] Monteiro HF, Faciola AP. 2020. Ruminal acidosis, bacterial changes, and lipopolysaccharides. J Anim Sci 98:skaa248. doi:10.1093/jas/skaa248 32761212 PMC7455920

[25] Sakai J, Cammarota E, Wright JA, Cicuta P, Gottschalk RA, Li N, Fraser IDC, Bryant CE. 2017. Lipopolysaccharide-induced NF-κB nuclear translocation is primarily dependent on MyD88, but TNFα expression requires TRIF and MyD88. Sci Rep 7:1428. doi:10.1038/s41598-017-01600-y 28469251 PMC5431130

[26] Castillo-Lopez E, Rivera-Chacon R, Ricci S, Reisinger N, Zebeli Q. 2022. Changes in fermentation profile of the reticulorumen and hindgut, and nutrient digestion in dry cows fed concentrate-rich diets supplemented with a phytogenic feed additive. J Dairy Sci 105:5747–5760. doi:10.3168/jds.2022-21786 35599024

[27] Jacobson NG, Szabo SJ, Weber-Nordt RM, Zhong Z, Schreiber RD, Darnell JE, Murphy KM. 1995. Interleukin 12 signaling in T helper type 1 (Th1) cells involves tyrosine phosphorylation of signal transducer and activator of transcription (Stat)3 and Stat4. J Exp Med 181:1755–1762. doi:10.1084/jem.181.5.1755 7722452 PMC2191986

[28] Yu LCH, Wang JT, Wei SC, Ni YH. 2012. Host-microbial interactions and regulation of intestinal epithelial barrier function: from physiology to pathology. World J Gastrointest Pathophysiol 3:27–43. doi:10.4291/wjgp.v3.i1.27 22368784 PMC3284523

[29] Dai H, Ma N, Chang G, Aabdin ZU, Shen X. 2022. Long-term high-concentrate diet feeding induces apoptosis of rumen epithelial cells and inflammation of rumen epithelium in dairy cows. Anim Biotechnol 33:289–296. doi:10.1080/10495398.2020.1806073 32808856

[30] Wang Y, Koroleva EP, Kruglov AA, Kuprash DV, Nedospasov SA, Fu Y-X, Tumanov AV. 2010. Lymphotoxin beta receptor signaling in intestinal epithelial cells orchestrates innate immune responses against mucosal bacterial infection. Immunity 32:403–413. doi:10.1016/j.immuni.2010.02.011 20226692 PMC2878123

[31] Pan XH, Yang L, Beckers Y, Xue FG, Tang ZW, Jiang LS, Xiong BH. 2017. Thiamine supplementation facilitates thiamine transporter expression in the rumen epithelium and attenuates high-grain-induced inflammation in low-yielding dairy cows. J Dairy Sci 100:5329–5342. doi:10.3168/jds.2016-11966 28501402

[32] Dinarello CA. 2000. Proinflammatory cytokines. Chest 118:503–508. doi:10.1378/chest.118.2.503 10936147

[33] Kent-Dennis C, Aschenbach JR, Griebel PJ, Penner GB. 2020. Effects of lipopolysaccharide exposure in primary bovine ruminal epithelial cells. J Dairy Sci 103:9587–9603. doi:10.3168/jds.2020-18652 32747102

[34] Zhang H, Niesel DW, Peterson JW, Klimpel GR. 1998. Lipoprotein release by bacteria: potential factor in bacterial pathogenesis. Infect Immun 66:5196–5201. doi:10.1128/IAI.66.11.5196-5201.1998 9784522 PMC108648

[35] Heumann D, Barras C, Severin A, Glauser MP, Tomasz A. 1994. Gram-positive cell walls stimulate synthesis of tumor necrosis factor alpha and interleukin-6 by human monocytes. Infect Immun 62:2715–2721. doi:10.1128/iai.62.7.2715-2721.1994 7516310 PMC302873

[36] Merlin T, Gumenscheimer M, Galanos C, Freudenberg MA. 2001. TNF-α hyper-responses to Gram-negative and Gram-positive bacteria in Propionibacterium acnes primed or Salmonella typhimurium infected mice. J Endotoxin Res 7:157–163. doi:10.1177/09680519010070021001 11521096

[37] Parameswaran N, Patial S. 2010. Tumor necrosis factor-α signaling in macrophages. Crit Rev Eukaryot Gene Expr 20:87–103. doi:10.1615/critreveukargeneexpr.v20.i2.10 21133840 PMC3066460

[38] Lin TL, Shu CC, Chen YM, Lu JJ, Wu TS, Lai WF, Tzeng CM, Lai HC, Lu CC. 2020. Like cures like: pharmacological activity of anti-inflammatory lipopolysaccharides from gut microbiome. Front Pharmacol 11:554. doi:10.3389/fphar.2020.00554 32425790 PMC7212368

[39] Lund PA, De Biase D, Liran O, Scheler O, Mira NP, Cetecioglu Z, Fernández EN, Bover-Cid S, Hall R, Sauer M, O’Byrne C. 2020. Understanding how microorganisms respond to acid pH is central to their control and successful exploitation. Front Microbiol 11:556140. doi:10.3389/fmicb.2020.556140 33117305 PMC7553086

[40] Coyte KZ, Schluter J, Foster KR. 2015. The ecology of the microbiome: networks, competition, and stability. Science 350:663–666. doi:10.1126/science.aad2602 26542567

[41] Vital M, Howe AC, Tiedje JM. 2014. Revealing the bacterial butyrate synthesis pathways by analyzing (meta)genomic data. MBio 5:e00889. doi:10.1128/mBio.00889-14 24757212 PMC3994512

[42] Besle J, Jouany J, Cornu A. 1995. Transformations of structural phenylpropanoids during cell wall digestion. FEMS Microbiol Rev 16:33–52. doi:10.1016/0168-6445(94)00058-7

[43] Russel JB. 2002. Rumen microbiology and its role in ruminant nutrition. Cornell University, Ithaca, USA.

[44] Patra A, Park T, Kim M, Yu Z. 2017. Rumen methanogens and mitigation of methane emission by anti-methanogenic compounds and substances. J Anim Sci Biotechnol 8:13. doi:10.1186/s40104-017-0145-9 28149512 PMC5270371

[45] de Lima Procópio RE, da Silva IR, Martins MK, de Azevedo JL, de Araújo JM. 2012. Antibiotics produced by Streptomyces. Braz J Infect Dis 16:466–471. doi:10.1016/j.bjid.2012.08.014 22975171

[46] Lima J, Auffret MD, Stewart RD, Dewhurst RJ, Duthie CA, Snelling TJ, Walker AW, Freeman TC, Watson M, Roehe R. 2019. Identification of rumen microbial genes involved in pathways linked to appetite, growth, and feed conversion efficiency in cattle. Front Genet 10:701. doi:10.3389/fgene.2019.00701 31440274 PMC6694183

[47] Liu J, Bian G, Sun D, Zhu W, Mao S. 2017. Starter feeding altered ruminal epithelial bacterial communities and some key immune-related genes’ expression before weaning in lambs. J Anim Sci 95:910–921. doi:10.2527/jas.2016.0985 28380582

[48] Penner GB, Beauchemin KA, Mutsvangwa T. 2006. An evaluation of the accuracy and precision of a stand-alone submersible continuous ruminal pH measurement system. J Dairy Sci 89:2132–2140. doi:10.3168/jds.S0022-0302(06)72284-6 16702280

[49] VDLUFA (Association of German Agricultural Analytic and Research Institutes). 2012. Die Chemische Untersuchung von Futtermitteln. VDLUFA-Verlag.

[50] Weiss WP, Tebbe AW. 2019. Estimating digestible energy values of feeds and diets and integrating those values into net energy systems. Transl Anim Sci 3:953–961. doi:10.1093/tas/txy119 32704859 PMC7200586

[51] Kononoff PJ, Heinrichs AJ, Buckmaster DR. 2003. Modification of the Penn State forage and total mixed ration particle separator and the effects of moisture content on its measurements. J Dairy Sci 86:1858–1863. doi:10.3168/jds.S0022-0302(03)73773-4 12778598

[52] Pacífico C, Ricci S, Sajovitz F, Castillo-Lopez E, Rivera-Chacon R, Petri RM, Zebeli Q, Reisinger N, Kreuzer-Redmer S. 2022. Bovine rumen epithelial miRNA-mRNA dynamics reveals post-transcriptional regulation of gene expression upon transition to high-grain feeding and phytogenic supplementation. Genomics 114:110333. doi:10.1016/j.ygeno.2022.110333 35278616

[53] Lladó S, Covino S, Solanas AM, Petruccioli M, D’annibale A, Viñas M. 2015. Pyrosequencing reveals the effect of mobilizing agents and lignocellulosic substrate amendment on microbial community composition in a real industrial PAH-polluted soil. J Hazard Mater 283:35–43. doi:10.1016/j.jhazmat.2014.08.065 25261758

[54] Probst M, Gómez-Brandón M, Bardelli T, Egli M, Insam H, Ascher-Jenull J. 2018. Bacterial communities of decaying Norway spruce follow distinct slope exposure and time-dependent trajectories. Environ Microbiol 20:3657–3670. doi:10.1111/1462-2920.14359 30003645

[55] Bolyen E, Rideout JR, Dillon MR, Bokulich NA, Abnet CC, Al-Ghalith GA, Alexander H, Alm EJ, Arumugam M, Asnicar F, et al.. 2019. Reproducible, interactive, scalable and extensible microbiome data science using QIIME 2. Nat Biotechnol 37:852–857. doi:10.1038/s41587-019-0209-9 31341288 PMC7015180

[56] Wei T, Simko V. 2017. R package “corrplot”: visualization of a correlation matrix. Version 0.84

[57] Martin M. 2011. Cutadapt removes adapter sequences from high-throughput sequencing reads. EMBnet J 17:10. doi:10.14806/ej.17.1.200

[58] Magoč T, Salzberg SL. 2011. FLASH: fast length adjustment of short reads to improve genome assemblies. Bioinformatics 27:2957–2963. doi:10.1093/bioinformatics/btr507 21903629 PMC3198573

[59] Callahan BJ, McMurdie PJ, Rosen MJ, Han AW, Johnson AJA, Holmes SP. 2016. DADA2: high-resolution sample inference from Illumina amplicon data. Nat Methods 13:581–583. doi:10.1038/nmeth.3869 27214047 PMC4927377

[60] Sabree ZL, Huang CY, Arakawa G, Tokuda G, Lo N, Watanabe H, Moran NA. 2012. Genome shrinkage and loss of nutrient-providing potential in the obligate symbiont of the primitive termite Mastotermes darwiniensis. Appl Environ Microbiol 78:204–210. doi:10.1128/AEM.06540-11 22020505 PMC3255634

[61] Dittmann KK, Rasmussen BB, Melchiorsen J, Sonnenschein EC, Gram L, Bentzon-Tilia M. 2020. Changes in the microbiome of mariculture feed organisms after treatment with a potentially probiotic strain of Phaeobacter inhibens. Appl Environ Microbiol 86:e00499-20. doi:10.1128/AEM.00499-20 32385083 PMC7357474

[62] McMurdie PJ, Holmes S. 2013. phyloseq: an R package for reproducible interactive analysis and graphics of microbiome census data. PLoS One 8:e61217. doi:10.1371/journal.pone.0061217 23630581 PMC3632530

[63] Ricci S, Pacífico C, Castillo-Lopez E, Rivera-Chacon R, Schwartz-Zimmermann HE, Reisinger N, Berthiller F, Zebeli Q, Petri RM. 2022. Progressive microbial adaptation of the bovine rumen and hindgut in response to a step-wise increase in dietary starch and the influence of phytogenic supplementation. Front Microbiol 13:920427. doi:10.3389/fmicb.2022.920427 35935232 PMC9354822

[64] Wickham H. 2016. ggplot2: elegant graphics for data analysis. Springer-Verlag, New York. https://ggplot2.tidyverse.org.

[65] Oksanen J. 2015. vegan: community ecology package. R Package Version 2.3-0. Available from: http://CRAN.R-project.org/package=vegan

[66] Mallick H, Rahnavard A, McIver LJ, Ma S, Zhang Y, Nguyen LH, Tickle TL, Weingart G, Ren B, Schwager EH, Chatterjee S, Thompson KN, Wilkinson JE, Subramanian A, Lu Y, Waldron L, Paulson JN, Franzosa EA, Bravo HC, Huttenhower C. 2021. Multivariable association discovery in population-scale meta-omics studies. PLoS Comput Biol 17:e1009442. doi:10.1371/journal.pcbi.1009442 34784344 PMC8714082

[67] Pang Z, Zhou G, Ewald J, Chang L, Hacariz O, Basu N, Xia J. 2022. Using MetaboAnalyst 5.0 for LC-HRMS spectra processing, multi-omics integration and covariate adjustment of global metabolomics data. Nat Protoc 17:1735–1761. doi:10.1038/s41596-022-00710-w 35715522

