# Supplementary material for: Dynamic interplay of immune response, metabolome, and microbiota in cows during high-grain feeding: insights from multi-omics analysis
Source: Microbiol Spectr. 2024 Aug 20;12(10):e00944-24. doi: 10.1128/spectrum.00944-24 (PMC11448160; doi:10.1128/spectrum.00944-24)
Supplement: Supplemental tables — Tables S1-S6. [file spectrum.00944-24-s0001.docx]

**Table S1**. Ruminal pH variations due to a dietary change to high-grain and the duration on this feeding regime in non-lactating Holstein cows.

|  | Duration on high-grain feeding^1^ | | | | |  |  |
| --- | --- | --- | --- | --- | --- | --- | --- |
| Item | Wk 0 | Wk 1 | Wk 2 | Wk 3 | Wk 4 | SE^3^ | *P*-value |
| Maximum pH | 6.8 | 6.62 | 6.76 | 6.79 | 6.78 | 0.04 | <0.01 |
| Minimum pH | 6.11 | 5.59 | 5.61 | 5.59 | 5.54 | 0.05 | <0.01 |
| Mean pH | 6.45 | 6.06 | 6.16 | 6.16 | 6.14 | 0.04 | <0.01 |
| Magnitude variation | 0.67 | 1.02 | 1.13 | 1.20 | 1.23 | 0.05 | <0.01 |
| Time <5.8, min/d^2^ | 1.62 | 228 | 147 | 138 | 214 | 53.8 | <0.01 |
| Area <5.8, min × pH^2^ | 0.19 | 33.4 | 34.9 | 21.8 | 44 | 0.49 | <0.01 |
| Acidosis index, time pH <5.8/kg DM^2^ | 0.13 | 18.6 | 14.1 | 12.3 | 18.8 | 6.59 | <0.01 |

^1^Wk 0 (week 0) corresponds to measures taken before the start of high-grain feeding.

^2^Because of a lack of normal distribution, data were subjected to square root transformation prior to statistical analysis, and then back-transformed.

^3^The largest standard error of the mean.

**Table S2.** Top ten correlation coefficients between the ruminal bacterial taxa and the genes associated with the NFkB pathway in non-lactating Hostein dairy cows used in the experiment.

| **Gene name** | **Bacterial taxa, correlation coefficient and *P*-value** | | | | | | | | | |
| --- | --- | --- | --- | --- | --- | --- | --- | --- | --- | --- |
| TNFR | Ruminococcaceae.UCG-005 | Mollicutes.RF39_metagenome_metagenome | F082_uncultured.rumen.bacterium | Corynebacterium.1 | Moryella | Atopobium | Uncultured.rumen.bacterium | Solobacterium | Fibrobacter | [Eubacterium].brachy.group |
|  | 0.548 | 0.472 | 0.448 | 0.430 | 0.415 | 0.408 | 0.359 | 0.356 | -0.344 | 0.331 |
|  | <.001 | <.001 | <.001 | <.001 | <.001 | <.001 | <0.001 | <0.001 | .001 | .002 |
| CD14 | Ruminococcaceae.UCG-005 | Candidatus.Stoquefichus | Mollicutes.RF39_metagenome_metagenome | Ruminococcaceae.UCG-002 | Corynebacterium.1 | Uncultured.rumen.bacterium | Uncultured.rumen.bacterium | Moryella | [Eubacterium].oxidoreducens.group | Pseudobutyrivibrio |
|  | 0.605 | 0.495 | 0.465 | 0.432 | 0.429 | 0.427 | 0.395 | 0.369 | 0.351 | -0.312 |
|  | <.001 | <.001 | <.001 | <.001 | <.001 | <.001 | <.001 | <.001 | .001 | .003 |
| NFkB | Uncultured.rumen.bacterium | Mollicutes.RF39_metagenome_metagenome | Uncultured.rumen.bacterium | Atopobium | [Eubacterium].oxidoreducens.group | Ruminococcaceae.UCG-002 | Ruminococcaceae.UCG-001 | GCA-900066575 | Unknown | [Eubacterium].brachy.group |
|  | 0.473 | 0.380 | 0.353 | 0.328 | 0.325 | 0.322 | 0.318 | -0.304 | -0.294 | 0.287 |
|  | <.001 | <.001 | <.001 | .002 | .002 | .002 | .003 | .004 | .006 | .007 |
| TLR4 | Porphyromonadaceae.bacterium | Atopobium | Gordonibacter | Erysipelotrichaceae.UCG-004 | Oscillospira | Ruminococcaceae.UCG-001 | Pygmaiobacter | Moryella | Caproiciproducens | Ruminococcus.1 |
|  | 0.577 | 0.496 | 0.440 | 0.424 | 0.381 | 0.375 | 0.317 | 0.302 | 0.294 | -0.266 |
|  | <.001 | <.001 | <.001 | <.001 | <.001 | <.001 | .003 | .004 | .006 | .013 |
| DSG1 | Dysgonomonadaceae_uncultured | Acetobacter | p-2534-18B5.gut.group_uncultured.bacterium | Lachnoclostridium.5 | Succiniclasticum | Mailhella | Erysipelotrichaceae.UCG-002 | Flavonifractor | Bilophila | Dorea |
|  | 0.501 | 0.501 | 0.500 | 0.477 | 0.457 | 0.454 | 0.443 | 0.431 | 0.423 | 0.418 |
|  | <.001 | <.001 | <.001 | <.001 | <.001 | <.001 | <.001 | <.001 | <.001 | <.001 |
| IL1R | Ruminococcaceae.UCG-005 | Lachnoclostridium.5 | BS11.gut.group_uncultured.bacterium | [Eubacterium].ruminantium.group | Peptococcaceae_uncultured | Coprococcus.3 | Ruminococcaceae.UCG-009 | Romboutsia | Dorea | Prevotellaceae.YAB2003.group |
|  | 0.482 | 0.444 | 0.408 | -0.405 | 0.378 | 0.377 | 0.377 | 0.372 | 0.370 | -0.368 |
|  | <.001 | <.001 | .001 | .001 | <.001 | <.001 | <.001 | <.001 | <.001 | <.001 |
| Ilb | uncultured.alpha.proteobacterium | vadinBB60.group_gut.metagenome | Lachnospiraceae.NK3A20.group | Acetitomaculum | Erysipelotrichaceae.bacterium | Lachnospiraceae.UCG-010 | Caproiciproducens | [Eubacterium].xylanophilum.group | uncultured.Parabacteroides.sp. | Ruminococcaceae_uncultured |
|  | 0.571 | 0.571 | 0.430 | 0.361 | 0.346 | 0.302 | 0.293 | -0.275 | 0.272 | 0.265 |
|  | <.001 | <.001 | <.001 | <.001 | .001 | .005 | .007 | .012 | .013 | .015 |

**Table S3.** Effect of duration of high-grain feeding on microbial metabolites from the ruminal fluid of non-lactating Holstein cows.

|  | Duration of the high-grain feeding^1^ | | | | |  |  |
| --- | --- | --- | --- | --- | --- | --- | --- |
| Item (mg/L) | Week 0 | Week 1 | Week 2 | Week 3 | Week 4 | SEM | *P*-value |
| **Carbohydrates and degradation derivatives** |  |  |  |  |  |  |  |
| Hexoses | 67.1b | 540a | 486a | 630a | 641a | 77.80 | <0.01 |
| Pentoses | 10.9b | 64.4a | 54.1a | 72.4a | 70.1a | 7.26 | <0.01 |
| Sucrose | 16.4b | 97.4a | 85.1a | 87.0a | 114a | 12.89 | <0.01 |
| Glucose 1-phosphate | 1.64b | 2.74b | 2.60b | 3.21ab | 4.52a | 0.36 | <0.01 |
| Glucose 6-phosphate | 3.06b | 6.07b | 13.9a | 15.1a | 18.9a | 2.35 | <0.01 |
| Mannose 6-phosphate | 1.16c | 1.36c | 2.82b | 3.61ab | 4.28a | 0.47 | <0.01 |
| Fructose 6-phosphate | 1.26b | 2.27b | 5.72a | 6.88a | 8.30a | 1.05 | <0.01 |
| Sedoheptulose 7-phosphate | 2.08c | 5.16b | 6.49b | 7.11a | 9.33a | 0.98 | <0.01 |
| Galactose 1-phosphate | 0.66b | 1.06b | 0.79b | 1.57a | 1.90a | 0.19 | <0.01 |
| Ribose 5-phosphate | 3.26b | 3.13b | 5.02a | 5.35a | 5.35a | 0.45 | <0.05 |
| Phosphoenolpyruvic acid | 0.87b | 0.76b | 0.74b | 1.69a | 1.89a | 0.23 | <0.01 |
| Phenylpropionic acid | 112a | 87.4b | 85.0b | 88.2b | 80.6b | 5.25 | <0.01 |
| Glyceric acid | 1.20c | 2.50ab | 2.26b | 2.83ab | 2.88a | 0.28 | <0.01 |
| Galacturonic acid | 1.33c | 3.00ab | 2.54bc | 3.64ab | 4.94a | 0.59 | <0.01 |
| Lactic acid | 0.13c | 0.88ab | 1.02a | 0.63b | 0.75b | 0.11 | <0.05 |
| Benzoic acid | 2.90a | 1.25b | 1.46b | 2.69a | 2.44a | 0.28 | <0.01 |
| Mannitol | 0.51 | 0.71 | 0.38 | 0.99 | 0.84 | 0.16 | 0.06 |
| Phosphoglyceric acid | 1.68b | 2.46b | 2.65b | 5.05a | 5.31a | 0.80 | <0.05 |
| **Aminoacid derivatives** |  |  |  |  |  |  |  |
| Phenylacetic acid | 17.0c | 15.0c | 24.3b | 32.7a | 31.3a | 2.95 | <0.01 |
| Pyroglutamate | 2.23 | 0.8 | 0.41 | 1.07 | 2.82 | 0.99 | 0.44 |
| **Short-chain fatty acids and related metabolites** |  |  |  |  |  |  |  |
| Acetic acid | 5016 | 5832 | 5325 | 5555 | 5316 | 307 | 0.36 |
| Propionic acid | 962b | 1273a | 1484a | 1434a | 1362a | 110.1 | <0.01 |
| Butyric acid | 970b | 1961a | 1879a | 1708a | 1602a | 144.9 | <0.01 |
| Isobutyric acid | 69.5 | 63.8 | 65.8 | 72.9 | 71.7 | 4.31 | 0.46 |
| Methylbutyric acid | 95.6 | 89.0 | 119 | 87.4 | 96.9 | 10.46 | 0.13 |
| Isovaleric acid | 74.7 | 64.1 | 72.5 | 76.5 | 81.0 | 5.57 | 0.21 |
| Valeric acid | 139c | 210a | 254a | 207ab | 192b | 17.0 | <0.01 |
| Hexanoic acid | 130.3a | 116ab | 81.7bc | 76.8c | 83.0bc | 13.0 | <0.05 |
| Hydroxyphenyl acetic acid | 2.50a | 1.29b | 1.52b | 2.33a | 2.28a | 0.23 | <0.01 |
| Methymalonic acid | 1.28 | 0.86 | 2.32 | 1.76 | 2.78 | 0.56 | 0.11 |
| Hydroxyphenyl propionic acid | 2.69 | 3.51 | 4.17 | 2.79 | 4.72 | 0.62 | 0.06 |
| **Citric acid cyle intermediates** |  |  |  |  |  |  |  |
| Citric acid | 0.16bc | 0.04c | 0.25bc | 0.64a | 0.54ab | 0.30 | 0.05 |
| Succinic acid | 1.57bc | 7.07b | 26.2a | 8.68ab | 22.1a | 6.42 | <0.05 |
| Alpha ketoglutaric acid | 0.92b | 0.75b | 0.69b | 1.12b | 1.57a | 0.17 | <0.01 |
| Fumaric acid | 0.96c | 0.82c | 1.12c | 1.28b | 1.74a | 0.14 | <0.01 |
| Malic acid | 1.93c | 3.44b | 4.42a | 4.92a | 5.35a | 0.48 | <0.01 |
| **Nucleotides** |  |  |  |  |  |  |  |
| Thymidine 5-monophosphate | 1.42c | 2.08b | 2.22b | 3.14a | 3.29a | 0.21 | <0.01 |
| Uridine 5-monophosphate | 1.91c | 1.94c | 2.72b | 3.57a | 3.48ab | 0.29 | <0.01 |
| Cytidine 5-monophosphate | 2.31c | 2.41c | 2.96bc | 3.45ab | 3.71a | 0.26 | <0.01 |
| Adenosine 5-monophosphate | 2.87c | 3.09c | 4.40bc | 6.43a | 5.70ab | 0.55 | <0.01 |
| Guanosine 5-monophosphate | 2.08b | 1.66b | 2.14b | 2.81a | 3.12a | 0.26 | <0.01 |
| **Biogenic Amines** |  |  |  |  |  |  |  |
| Alpha-aminobutyric acid | 0.90 | 2.01 | 3.07 | 1.40 | 2.11 | 0.81 | 0.50 |
| Aminovaleric acid | 1.37 | 9.15 | 7.99 | 4.98 | 6.16 | 3.01 | <0.01 |
| Beta-alanine | 0.53 | 0.95 | 0.71 | 0.48 | 0.60 | 0.18 | 0.50 |
| Cadaverine | 3.05 | 3.57 | 3.63 | 3.05 | 3.58 | 0.29 | 0.50 |
| Ethanolamine | 1.33 | 2.61 | 2.39 | 2.40 | 2.23 | 0.50 | 0.40 |
| Gamma-aminobutyric-acid | 1.11 | 3.56 | 7.51 | 1.47 | 5.52 | 2.71 | 0.60 |
| Histamine | 0.08 | 0.25 | 0.25 | 0.19 | 0.23 | 0.07 | <0.05 |
| Putrescine | 1.76 | 4.19 | 5.04 | 4.37 | 4.71 | 1.30 | <0.01 |
| Phenylethylamine | 0.95 | 0.70 | 0.67 | 0.91 | 1.28 | 0.25 | 0.50 |
| Pyrrolidine | 4.49 | 4.13 | 4.50 | 4.60 | 3.72 | 0.36 | 0.50 |
| Spermidine | 2.86 | 6.56 | 6.54 | 5.93 | 5.49 | 1.53 | <0.05 |
| Spermine | 1.66 | 3.31 | 2.82 | 2.72 | 2.48 | 0.61 | 0.60 |

^1^Week 0 represents a week when a forage diet was offered. a,b,c: Within each row, different letters indicate a significant difference.

**Table S4.** Feed intake of each of the cows used in the experiment, values are the average within each feeding week for both experimental periods.

| Cow number | Feeding week | Diet | Dry matter intake, kg/d |
| --- | --- | --- | --- |
| One | 0 | Forage | 8.12 |
| One | 1 | High-grain | 9.82 |
| One | 2 | High-grain | 9.91 |
| One | 3 | High-grain | 10.1 |
| One | 4 | High-grain | 10.9 |
| Two | 0 | Forage | 10.0 |
| Two | 1 | High-grain | 13.3 |
| Two | 2 | High-grain | 12.4 |
| Two | 3 | High-grain | 12.8 |
| Two | 4 | High-grain | 13.4 |
| Three | 0 | Forage | 10.5 |
| Three | 1 | High-grain | 13.4 |
| Three | 2 | High-grain | 8.79 |
| Three | 3 | High-grain | 9.57 |
| Three | 4 | High-grain | 9.53 |
| Four | 0 | Forage | 10.5 |
| Four | 1 | High-grain | 13.8 |
| Four | 2 | High-grain | 9.93 |
| Four | 3 | High-grain | 10.7 |
| Four | 4 | High-grain | 10.0 |
| Five | 0 | Forage | 11.8 |
| Five | 1 | High-grain | 11.7 |
| Five | 2 | High-grain | 7.85 |
| Five | 3 | High-grain | 8.96 |
| Five | 4 | High-grain | 11.4 |
| Six | 0 | Forage | 8.36 |
| Six | 1 | High-grain | 10.3 |
| Six | 2 | High-grain | 10.0 |
| Six | 3 | High-grain | 9.55 |
| Six | 4 | High-grain | 9.07 |
| Seven | 0 | Forage | 12.1 |
| Seven | 1 | High-grain | 13.9 |
| Seven | 2 | High-grain | 12.7 |
| Seven | 3 | High-grain | 12.2 |
| Seven | 4 | High-grain | 10.9 |
| Eight | 0 | Forage | 9.83 |
| Eight | 1 | High-grain | 12.0 |
| Eight | 2 | High-grain | 10.7 |
| Eight | 3 | High-grain | 11.6 |
| Eight | 4 | High-grain | 12.4 |
| Nine | 0 | Forage | 10.6 |
| Nine | 1 | High-grain | 14.7 |
| Nine | 2 | High-grain | 13.0 |
| Nine | 3 | High-grain | 13.3 |
| Nine | 4 | High-grain | 13.2 |

**Table S5.** Ingredients, chemical composition, and particle size distribution of the diets fed to non-lactating Holstein cows during the week of forage feeding and during the 4 weeks of high-grain feeding.

| Item | Forage diet | High-grain diet |
| --- | --- | --- |
| Ingredients, % DM |  |  |
| Grass hay | 10.0 | 0 |
| Grass silage | 45.0 | 26.3 |
| Corn silage | 45.0 | 8.75 |
| Concentrate^1^ | 0 | 65.0 |
| TMR chemical composition |  |  |
| DM, % as fresh | 34.0 | 47.2 |
| Crude protein, % | 11.5 | 17.6 |
| Neutral detergent fiber (NDF), % | 55.5 | 31.6 |
| Acid detergent fiber (ADF), % | 34.2 | 21.5 |
| Starch, % | 17.0 | 28.7 |
| Ether extract, % | 1.98 | 2.78 |
| Non-fiber carbohydrates, % | 22.9 | 40.5 |
| Residual organic matter, % | 5.9 | 11.8 |
| Ash, % | 6.70 | 6.72 |
| Particle fraction (% retained)^2^ |  |  |
| Long | 64.5 | 29.1 |
| Medium | 21.3 | 30.1 |
| Short | 13.6 | 38.8 |
| Fine | 0.51 | 1.90 |

^1^The pelleted concentrate mixture contained: wheat (30.36%), triticale (18.06%), bakery by-product (23.02%), rapeseed meal (23.94%), molasses (2.99%), mineral-vitamin premix for dairy cattle (1.53%) and limestone (1.0%).

^3^Particle fractions determined by Penn State Particle Separator with a 19-mm screen (long), 8-mm screen (medium), 1.18-mm screen (short), and a pan (fine) according to Kononoff et al. (2003).

**Table S6.** Primers used for gene expression analysis of the NF-kB pathway and tight junction encoding genes.

| Gene | Forward primer | Reverse primer | Amplicon (bp) | Annealing temp. (°C) | Accession number | Reference |
| --- | --- | --- | --- | --- | --- | --- |
| NFkB | ATACGTCGGCCGTGTCTAT | GGAACTGTGATCCGTGTAG | 144 | 58 | NM_001076409.1 | Jin et al. 2016 |
| IL1β | CATGTGTGCTGAAGGCTCTC | GATACCCAAGGCCACAGGAA | 119 | 60 | NM_174093.1 | Petri et al., 2019 |
| IL1R | CACACACTTGGGAAAGCAGT | TCGACCCATTCCACTTCCAA | 197 | 63 | ENSBTAG00000005273 | This study |
| IL12A | CTGCCTCGACTACTCCCAAA | TGGAGGCCTGTTTACCACTG | 157 | 57 | ENSBTAG00000015150 | This study |
| IL6 | CACCCCAGGCAGACTACTTC | GCATCCGTCCTTTTCCTCCA | 184 | 62 | ENSBTAG00000014921 | Ricci et al., 2023 |
| IL8 | GGAAAAGTGGGTGCAGAAGG | CTACACCAGACCCACACAGT | 186 | 63 | ENSBTAG00000019716 | This study |
| TNFα | AGCCCTCTRGTTCARACACT | GCTGGTTGTCTTCCAGCTTC | 159 | 63 | ENSBTAG00000025471 | Hartinger et al., 2023 |
| TNFR2 | AGCAGCACGGACAAGAGG | TTGACAATGCAGGTGACGTT | 155 | 58 | AF031589.1 | This study |
| LTB | GGGAGGATTGGTGAGTGAGT | AAACGCCTCTTCTTTCTTCG | 145 | 57 | ENSBTAG00000020674 | This study |
| CD14 | ATCCACAGTCCAGCCGACAA | CAGCAGCAGCAGCAGGTAGG | 97 | 60 | NM_174008.1 | Petri et al., 2019 |
| MYD88 | GACGACGTGCTGATGGAACT | CCGGATCATCTCGTGGACAA | 111 | 60 | ENSBTAG00000000563 | Ricci et al., 2023 |
| TLR4 | TGGGACCCTTGCGTACAG | ACGGCCACCAGCTTCTG | 159 | 60 | XM_005210586.3 | Ricci et al., 2023 |
| Housekeeping genes^1^ | |  |  |  |  |  |
| HPRT1 | TTGTATACCCAATCATTATGCTGAG | ACCCATCTCCTTCATCACATCT | 109 | 58 | NW_005397637.1 | Ricci et al., 2024 |
| OAZ1 | CACAAGAACCGTGATGATCGA | TCTCACAATCTCAAAGCCCAAA | 69 | 58 | NM_001127243.2 | Petri et al. 2018 |
| YWHAZ | TGAAAGGAGACTACTACCGCTACTTG | GCTGTGACTGGTCCACAATCC | 121 | 58 | NM_174814.2 | Ricci et al., 2023 |

^1^HPRT1: Hypoxanthine phosphoribosyltransferase 1; OAZ1: Ornithine decarboxylase antizyme 1; YWHAZ: 14-3-3 protein zeta/delta.
